# A pilot randomised controlled trial of a critical time intervention for people leaving prison: findings from an integrated process evaluation

**DOI:** 10.1136/bmjopen-2024-097761

**Published:** 2025-12-17

**Authors:** Adam Dale Newman Williams, Nina Jacob, Yvonne Moriarty, Iolo Madoc-Jones, Suzanne Fitzpatrick, Peter Mackie, Ian Thomas, Detelina Grozeva, Barry Lloyd, Manuela Deidda, Samuel Owusu Achiaw, Kelly Lewis, Rebecca Cannings-John, Srinivasa Vittal Katikireddi, James White, Jim Lewsey

**Affiliations:** 1 Cardiff University, Cardiff, UK; 2 Centre for Trials Research, Cardiff University, Cardiff, UK; 3 Wrexham University, Wrexham, UK; 4 Heriot-Watt University, Edinburgh, UK; 5 School of Geography and Planning, Cardiff University, Cardiff, UK; 6 Health Economics and Health Technology Assessment, University of Glasgow, Glasgow, UK; 7 University of Glasgow, Glasgow, UK; 8 MRC/CSO Social & Public Health Sciences Unit, University of Glasgow, Glasgow, UK

**Keywords:** QUALITATIVE RESEARCH, Vulnerable Populations, Prisons, Feasibility Studies

## Abstract

**Background:**

We conducted a pilot randomised controlled trial (the PHaCT study), including a process evaluation to assess the acceptability of a housing-led Critical Time Intervention (CTI) for prison leavers and the use of a trial design. This paper presents the process evaluation findings.

**Objective:**

To explore the acceptability of both the intervention and the trial design to participants and those delivering the intervention, and to assess whether the intervention was delivered with fidelity.

**Design:**

A process evaluation following Medical Research Council guidelines. Data collection included semi-structured interviews with participants and CTI caseworkers and observations of intervention delivery. A thematic analysis of interviews and observations was conducted to understand the intervention’s implementation and contextual factors as well as the trial process acceptability.

**Setting:**

Participants for the pilot trial were recruited from three prisons in England and Wales where the intervention was being delivered.

**Participants:**

While 28 out of 34 trial participants consented to interviews, only one was completed. Seven caseworkers were interviewed.

**Intervention:**

A housing-led CTI to support people leaving prison at risk of homelessness, involving phased, time-limited support from caseworkers, starting prerelease and continuing postrelease, to help secure stable housing and build independence, without directly providing housing.

**Results:**

The intervention’s acceptability was primarily reflected through the positive feedback and success stories shared by CTI caseworkers, as well as observational data indicating high acceptance among service users. The trial design’s acceptability was challenged by concerns about randomisation and equipoise, with staff viewing randomisation as unethical due to limited support for vulnerable populations. The fidelity to the CTI intervention housing-led approach was adhered to as best as possible; stable housing was prioritised for service users before addressing other needs. Despite these efforts, both sites encountered significant challenges due to limited housing availability and complex systems for securing social housing, particularly for single men leaving prison.

**Conclusions:**

This wider study faced significant challenges which impacted the process evaluation. Despite these issues, the evaluation provides important insights into the challenges of conducting trials on interventions for people leaving prison. The challenges experienced should inform future study designs with similar populations and in similar settings.

**Trial registration number:**

ISRCTN46969988.

**STRENGTHS AND LIMITATIONS OF THIS STUDY**

- The use of multiple qualitative methods, including interviews, observations and field notes, enabled triangulation of data sources to enhance the credibility of findings.
- Fidelity to the intervention was assessed using a structured framework adapted from existing critical time intervention fidelity tools, allowing for a systematic evaluation across sites.
- The trial faced significant recruitment and retention challenges, particularly in engaging participants postrelease, which limited the ability to collect direct feedback from service users.
- The use of standard clinical research recruitment methods, rather than a dedicated, embedded research team with experience in prison settings, may have contributed to low follow-up rates and reduced participant engagement.

## Introduction

 People released from prison struggle to reintegrate into their communities and often face a high risk of homelessness. Nearly one in three people released from prison in England and Wales lack a settled home and remain homeless or in unstable housing a year later.^1^ This is often due to challenges such as a lack of suitable affordable housing, the absence of a stable support network and unmet support needs linked to substance use and mental ill health.^2^,^6^ Individuals without stable housing experience lower life expectancy and worse quality of life.^7^ People experiencing homelessness are at a higher risk than the general population of infectious and non-communicable diseases,^3^ mental health problems, alcohol and substance use,^4^ have higher rates of emergency hospital admissions^5^ and report lower levels of well-being and health-related quality of life.^6^

Critical time interventions (CTIs) aim to support vulnerable individuals during significant life transitions, such as leaving inpatient psychiatric care, homeless shelters or prisons. CTIs are time-limited and aim to improve an individual’s engagement with treatment and community services through developing problem-solving skills.^8^,^10^ Homelessness services suggest that CTIs work best when they are ‘housing-led’, meaning housing is accessed quickly with minimal preconditions.^8^ Most evidence for CTI originates from the USA, with few studies originating in the UK and many focusing on mental health outcomes.^8 9^ No studies have evaluated how effective and cost-effective housing-led CTIs are in preventing homelessness and/or improving the health of people leaving prison.

### Housing-led CTI

A UK-based charity developed a CTI model to support people leaving prison at risk of experiencing homelessness. The specific CTI model emphasised a ‘housing-led’ principle which prioritises securing stable housing for service users before addressing other wider social or health needs.^10^ This model begins with the CTI caseworker engaging with the service user in prison to develop a rapport and begin the transition process. After the service user is released from the institution, they are supported to gain a tenancy (immediately or following a brief period in temporary accommodation). The support follows three phases (lasting 3 months each):

1. Phase 1 (‘transition to the community’): a period of forming links and relationships and shared goal setting; aiming to improve crisis-resolution skills, provide support and advice tailored to needs, and mediate any conflicts. This phase involves weekly home visits and other meetings with the service user, any caregivers and community service providers.
2. Phase 2 (‘try out’): involves fewer meetings as the caseworker, with the help of community resources and family members, encourages the service user to independently problem-solve and manage practical issues (eg, sorting bills and general money/benefits management; living skills; social support and meaningful use of time; managing housing provider relationship). At this point, the caseworker intervenes assertively only if the service user is receiving inadequate support or if a crisis occurs.
3. Phase 3 (‘transfer of care’): support reduces, the caseworkers help the service user to develop a plan to achieve long-term goals (eg, employment, family reunification) and finalise the transfer of responsibilities to any caregivers and community providers (ie, the CTI intervention is terminated with support in place).

This intervention does not provide housing directly, nor does the charity have a stock of properties available. Without privileged or enhanced access to housing stock, the model’s approach relies on referral, advocacy and support. The intervention was delivered by CTI caseworkers who work for the host charity (specific teams focused on supporting those leaving prison to secure housing) based at two locations, one in Wales (site 1) and one in England (site 2). Referrals were made to the CTI team from probation officers and resettlement teams within the prisons. The intervention was funded by the UK charity who were delivering it.

A pilot study was conducted to evaluate the feasibility of using a randomised controlled trial (RCT) design to assess the effectiveness of this housing-led CTI. The integrated process evaluation aimed to:

- explore the acceptability of the intervention to participants and those delivering the intervention.
- explore the acceptability of the trial design to participants and those delivering the intervention.
- to assess if the intervention was delivered with fidelity.

For context, the trial was a parallel two-arm, individual-level RCT of a pre-existing CTI intervention with an integrated process evaluation and embedded exploratory health economic evaluation. Site 1 (Wales) received referrals related to people from a single prison in Wales. Site 2 (England) was meant to receive referrals from three prisons in England, but in practice, only two of these prisons were regularly providing referrals. Participants were to be followed up in the community at both sites. The locations were predetermined by where the intervention was already being delivered by the intervention provider (CTI teams).

Participants were eligible for the trial if they met all inclusion criteria and none of the exclusion criteria set by the CTI team. Eligible individuals were aged 18 years or over; were due to be released into the local authority areas covered by the intervention delivery team; were able to access benefits and local authority housing assistance and had experienced homelessness at least once. Individuals were excluded if they were deemed at high risk of causing serious harm to others by probation and/or were eligible for or receiving Housing First (ie, support needs too high/complex to benefit from CTI).

The full description of the pilot study is provided in a separate full trial results paper.

## Methods

### Design

The process evaluation was designed following the Medical Research Council guidelines for evaluating complex interventions.^11^ The evaluation aimed to assess the acceptability of the intervention and trial processes, the fidelity of the intervention implementation, with consideration to contextual factors influencing the outcomes of the intervention.

### Patient and public involvement

Patients and the public were involved in the PHaCT study during the early stages of research design. An individual with lived experience of homelessness contributed to the design phase when applying for funding. The research questions were developed to align with the needs and experiences of the population at risk of homelessness. Patient and public involvement assisted in the development of study materials.

### Data collection and procedures

#### Interviews

Semistructured interviews were to be conducted with participants and CTI caseworkers from both England and Wales. The intention was to conduct 40 interviews with 12 participants from the intervention group (12 participants interviewed twice, once at phase 1 and another at phase 3 of the intervention) and 16 interviews with participants from the control group. Interviews were to take place in the community where phases 1–3 occurred. In the pilot trial, 34 participants were recruited out of a planned 80, and 28 of the 34 (82%) consented to be interviewed and observed at later stages in the intervention. However, only one trial participant was able to be interviewed. The study faced significant challenges maintaining contact with the participants after their release from prison, which made contacting participants for interview difficult. Seven CTI caseworkers were interviewed, out of a possible nine across both sites.

The interview schedules guided discussions on mapping the overall system (the criminal justice system, support on release from prison and relation to homelessness), theorising the CTI approach, and discussion of the RCT design. Interviews were conducted either in the CTI offices or via secure video conferencing software (Teams), whichever was most convenient for participants. All interviews were between October 2023 and August 2024. Interviews were audio recorded and transcribed verbatim by a Cardiff University-approved transcription supplier.

#### Observations

24 observations were planned with participants, 12 from the intervention group and 12 from the control group. However, due to difficulties at the initiation of the project, we expanded the observations to include service users of intervention not enrolled as participants, that is, those who started receiving support before the study randomisation began. These ranged across the phases of the intervention. Of the intended 24 interviews with participants, only eight occurred, most of these occurring at the prerelease phase of the intervention due to the difficulties of engaging RCT participants at follow-up. The rest of the observations occurred with non-RCT service users and ranged across the prerelease stage and three phases of the intervention. The observations occurred at the prisons and CTI offices in England and only the CTI offices in South Wales. Field notes were maintained throughout the study, compiled using deidentified information from observations, emails and informal discussions conducted between the researcher and all CTI staff during the period when the trial was being delivered.

### Lived experience engagement

An individual with lived experience of homelessness contributed to the design phase when applying for funding, attending trial management group meetings and to inform and review the research tools. The research questions were developed to align with the needs and experiences of the population at risk of homelessness.

### Analysis

Two researchers independently read the transcripts and familiarised themselves with these, along with the observation notes and other field notes. Qualitative data obtained from these three sources were analysed using thematic analysis. Data from all three sources—researcher field notes, interview transcripts and observation notes—were triangulated to gain a multilayered understanding of the findings.

These data were separated into individual response items and managed using NVivo V.12.^12^ The first researcher examined the data and coded each item, employing open coding. Several iterations of grouping and regrouping took place to fit all items into identified codes. These coded data were then examined to identify candidate themes. Each candidate theme was re-examined to ascertain if it accurately described the data collected and if all coded data were captured within these identified candidate themes. Quotes are used to illustrate the findings.

The second researcher independently examined the coded data and also identified candidate themes. These two researchers then compared and discussed the coding and resultant themes, moving back and forth within the data to ensure that the themes captured the meaning of the coded data. They revised the fit of the coded data into each theme where necessary. The wider research team, who were not involved in data collection, were invited to scrutinise the data and arbitrate any differences between findings. This method of analysis provided researcher triangulation, aiming to obtain a broader picture of the data.

Fidelity to the CTI model was measured against five fidelity items: housing-led approach, time-limited and phased approach, caseloads and supervision, person-centred approach/community focused and harm reduction/recovery-orientated approach. These items were informed by previous fidelity measures of CTI^13^ and discussions with the CTI caseworkers to ensure the measures reflected the housing-led model the charity set out to deliver. The two data collectors reviewed the interviews and observational data and synthesised the narrative behind each fidelity item from the qualitative data available.

### Challenges experienced

The trial faced significant delays affecting recruitment timelines, starting with approval and contractual issues. Initially planned to open within 6 months, site 1 (Wales) began recruiting after 16 months, and site 2 (England) after 22 months. Delays included prolonged approval and contractual processes and intervention staff shortages which led to changes throughout the study. The Probation Service faced severe staffing shortages and high caseloads, which affected its ability to support the study. The focus on managing high-risk offenders and the introduction of the End of Custody Supervised License scheme (ECSL) and Probation Reset schemes further strained resources.^14 15^ These constraints often resulted in reduced contact between probation practitioners and individuals leaving prison, limiting the support available to CTI participants. The unpredictability of early releases under the ECSL scheme also disrupted the planned intervention timeline, making it difficult for caseworkers to provide consistent support, as well as challenging the delivery of the pilot trial. This impacted the ability to collect primary data for the process evaluation from those receiving the intervention.

## Results

Trial recruitment faced many challenges and from the intended 80 participants, 34 individuals were recruited from three prisons (43% of the target). Of the 34 participants recruited, there were three withdrawals, leaving 31 participants, of which only six participants were successfully followed up (19%), four from the intervention group and two from the control group. In the intervention arm (n=17), six did not have a probation officer (attending probation meetings was the main method for following up participants), five participants were recalled to prison and five disengaged from the intervention. In the control group (n=14), five did not provide any contact information for follow-up, three returned to prison, six did not have a probation officer assigned and there was a death reported.

### Acceptability of the intervention

Due to the difficulties following up with participants, only one participant, who was in the control group, was interviewed. Thus, reflections on the intervention acceptability only come from the staff who deliver the intervention and the observations of the intervention delivery. We recognise important participant perspectives are missing from this analysis.

Early in the evaluation, it became evident that the CTI was highly valued by the CTI caseworkers, who shared numerous success stories directly attributed to the intervention. The widespread acceptance of CTI later introduced challenges related to the trial methodology, particularly concerning the acceptability to the CTI caseworkers of random allocation. This is likely similar to other social policy interventions, where those delivering it will in most cases view the intervention as generating some positive outcome. Naturally, the intervention was linked to their employment, introducing a potential bias in the CTI caseworker’s perspective when delivering the intervention.

I do firmly believe it works. I think housing-wise because of the state of the market, it’s not as successful KPI-wise on paper as I’d like it to look. But I think the softer outcomes outweigh that every time, like having people feel supported, feeling like there are people there for them. I think that motivates people to stop reoffending. PID 102, caseworker, site 1

Observational data highlighted that many service users expressed a high level of acceptance of the CTI model. Service users who were housed expressed gratitude for the support provided. The phased approach was deemed appropriate as service users became settled, expressing their agreement with having less contact once settled. However, a minority of service users challenged the provision of CTI, desiring greater support than the model provided and preferring to be given accommodation rather than searching for it themselves.

### Acceptability of the trial design

The trial design adopted individual-level randomisation for statistical efficiency. Although the research team considered a cluster-randomised design, it was not chosen due to concerns about contamination between clusters and that at full-scale trial the sample size would require much larger numbers to be adequately powered.

As mentioned, the intervention was developed by a third-sector organisation, adapted from previous CTI models and was already being delivered before a trial was proposed. This is different to usual practice or other trials where the intervention being provided is a new offer. The study was developed in collaboration with leaders at the organisation who maintained their support throughout the project. However, early in set-up, the teams delivering the intervention raised concerns about the principle of randomisation and as the CTI caseworkers believed the intervention was already effective, the principle of equipoise was challenged. Intervention teams were engaged and supported to understand the rationale for the study with multiple discussions being held and strong working relationships formed.

Despite agreeing to continue participation in the trial, many caseworkers viewed randomisation as unethical as it would withhold the intervention from such a vulnerable population, especially in light of the limited alternative support available. The stretched prison services, combined with the scarcity of any additional support, compounded the issue, further fuelling concerns about fairness. Staff members expressed this sentiment, stating that it was ‘cruel’ to deny the intervention due to the limited alternative sources of support: ‘It’s either the CTI or nothing’. The intervention team had limited resources, so they could not provide the service to everyone. However, to deny support when they did have capacity was very difficult for staff members who were focused on supporting people. Of course, the same number of people could still receive support, but randomisation meant it was not the usual first come, first served approach.

… we’re dealing with people and not just people but people’s lives and if they don’t have support coming out of prison this might be the time that they take too many drugs and they die, and I don’t feel comfortable being any contributing factor to that. PID 106, caseworker, site 2

Concerns about equipoise varied as a function of what usual care was provided. In site 1, where concerns about the trial methodology were most pronounced, control group participants were only provided with a list of alternative services, including contact information for the organisation’s wider services, which was available to all people at risk of homelessness, not just prison leavers.

In contrast, site 2 control group participants were enrolled on an alternative programme based within the community providing 6 months of support as opposed to the 9 months provided by the study intervention. Early discussions highlighted that defining what constituted as ‘usual care’ would be problematic between the sites as available services differed. However, ethically, services available in site 2 (but not site 1) could not be denied to participants in the study. As one respondent put it:

If they’re on [alternative model], then they’re getting virtually the same services as what I’m giving [in the intervention group], but just, we wait until you get out, and then we start and it’s only six months. PID 101, caseworker, site 2

Site 2’s usual care was more significant than in site 1. Although within a full-scale trial, this would add complexity to distinguishing the specific effects of the intervention. The provision of the alternative model to the usual care group ultimately enhanced the acceptability of the randomisation process for staff at site 2. In a full-scale RCT, the meaning of ‘usual care’ would require careful definition, as significant variation in the availability and uptake of services among control group participants could undermine the validity of the findings. Given that the two sites for this study were located in England and Wales, however, countries with distinct social policies and legislative frameworks relating to housing and support services, establishing the commonalities around usual care would be problematic.

Some CTI caseworkers expressed concern about the ability of participants to fully understand the nature of the study. There were concerns raised about obtaining informed consent due to the vulnerability of the prison population.

But the reality of the population here, they don’t know what a randomised control trial is. They don’t understand what research is. And a lot of them have got like, a lot of them didn’t go to school, a lot of them can’t read and write. It’s not really realistic to have that kind of in-depth conversation. PID 103, caseworker, site 1

This was coupled with the fact that consent was being sought within the prison environment. The issue of coercion within prison research has long been written about,^16^ and within the current study, concerns were raised among the research team and by CTI caseworkers about the extent to which potential participants agreed to take part out of desperation rather than fully understanding or agreeing to the nature of the research.

I’m not sure they fully understand what they’re consenting to. And I don’t think it’s a capacity thing. I don’t think explaining it anymore could actually make a difference; I just think it’s not their priority. Erm, and they’re so desperate, they’ll agree to anything that they think might help them. PID 102, caseworker, site 1

Recruitment and data collection were completed by research nurses (who received training from the research team) or a member of the research team. A randomisation script was followed, this being developed with input from the CTI teams. The research nurses and team members who completed recruitment and data collection felt that the individuals recruited comprehended the study and randomisation process sufficiently to participate in the study. However, this contradicted the CTI caseworkers who indicated that some participants struggled to accept concepts like random allocation and research in general.

I’ve had one guy who is actually in the intervention group now, he kept saying to me how do I get into the group with your support and I was like, it’s randomised, there’s nothing you can do and he’s like what shall I say in the answers, and I’m like, it’s randomised, there’s nothing, can you put a good word in for me and I was like, it’s not up to me, I’m really sorry. You go in and you tell the truth and then you find out. PID 104, caseworker, site 1

However, the confusion or lack of acceptance in this case may be due to the continued exposure to the intervention team. Usually in trials, the participants would only meet the intervention teams after being randomised, and the control group would not meet the intervention staff or have very limited interactions. In this case, the intervention team were already known from their regular attendance at the prison including after randomisation in the prison before release or in the community after release.

### Fidelity to the intervention

#### Housing-led approach

The housing-led CTI prioritises securing stable housing for service users before addressing other wider social or health needs. In adherence to this, CTI caseworkers prioritised finding stable housing for service users from the outset. Housing was a focus of all conversations observed, from the first meeting with service users in prison during the pre-CTI period to all subsequent follow-up appointments where housing was yet to be secured.

To be true to a housing-led approach is to say that priority should be given to enabling people to access settled housing as quickly as possible. For site 1, supporting people to gain a tenancy was a major focus, but the dire state of site 1’s housing market meant that individuals could not have immediate or speedy access to housing. Thus, while the CTI team adhered to the housing-led philosophy, the structural context made delivery problematic.

Because we are in such a state obviously like everywhere with housing. Nine months is not a long time to get accommodation. So, it’s making them feel settled where they are if it’s in temp and then if there’s any other groups in the community which they are interested in.” PID 5, caseworker, site 1

CTI caseworkers focused on ensuring that potential housing was suitable for everyone, at times going to great lengths to make sure that, if abstinence was important, then a service user was not housed somewhere where substance misuse was common.

You’ve got to be pretty resilient with it as well because his first week was chaos. He came out, he was placed in temporary accommodation out of the area. He was in [City], then he was placed somewhere in [town], which doesn’t have a great reputation and he was really struggling there, everyone was using, so he, he wasn’t staying there, but then probation had said that he needed to and he’d be recalled if he didn’t, so we were on the phones for hours trying to get somewhere different and then negotiate with probation. PID 102, caseworker, site 1

For site 2, housing was prominent from the beginning of the intervention and moving individuals into their own, private rented accommodation was a priority. The focus of initial discussions centred on making sure someone has the resources available to sustain a tenancy.

Let’s go through your incomings and outgoings. We’ll go through it as if you were going for a tenancy stay normally, just so you know where you stand. We will fill this in so my manager can have oversight of it as well, just so we can be sure of affordability. We’ll go through your income. What do you think you’ll be spending on gas and electricity [CTI worker then goes through the incoming form together detailing expected spending each month on utilities, food, entertainment etc.] Observation, site 2

While the housing market in site 2 was also challenging, there was greater availability of suitable properties. Observations at site 2 revealed a close connection between housing providers and CTI caseworkers throughout the area, with the two in regular contact about newly available homes. This resulted in faster results in getting individuals into settled accommodation, with site 2 able to provide costs to cover the bond and first month’s rent for new tenants as well. Staff at site 2 were also able, on occasion, to cover or pay off debts that accrued to support service users to ensure their benefits could be maintained and assist with rental payments to retain tenancies during short prison recalls. Due to having greater access to housing, income and finances were the most pressing concern at site 2. As one case worker explained, *“Affordability is a big one … if you’re only in receipt of basic Universal Credit, of two hundred and eighty pounds a month, you’re not affording a tenancy” PID 106, caseworker, site 2*

Discussions centred on maximising income for service users, to increase the likelihood of them affording a tenancy. This could be through support to complete benefit claims or exploring employment. Property searching and equipping service users with the skills to find suitable accommodation were key focuses of observed meetings. As one caseworker described,

We property search in most of the support sessions, in your pre-stage, then they’re property searching as well afterwards … and we’ll sit and go, right, okay, where are we looking for? How much are we looking at? … As long as they’re continuing to keep a bit of momentum, that’s the most important thing. PID 106, caseworker, site 2

Additionally, caseworkers contacted property providers or agencies on behalf of the service user to arrange viewings and prepare service users by suggesting possible questions to ask during the viewing. CTI workers attended viewings with the service user, offering them guidance on what to wear and how to present themselves. Once a suitable property was found, they assisted in completing application forms, often using prepared statements to ensure consistency.

Without exception, the caseworkers identified challenges associated with the availability and standard of housing, with the absence of suitable properties being the principal barrier to the operation of the CTI. There were also reports of the statutory homelessness system being complex to navigate so that a large part of the caseworker’s role was about assisting service users to navigate their way through it, especially in the early days post-release from prison.

The system is really difficult to navigate. And people that have been in and out loads of times, they still don’t understand it either. So sometimes we’d have someone who’s like a prolific offender, and they still don’t understand they’ve got to go to Housing Options on release. So, they’re like ‘Oh are you going to find me somewhere?’ PID 103, caseworker, site 1

Due to CTI service users being mostly single men, they were given lower priority when it came to securing social housing and so their transition into settled accommodation became difficult:

The housing bit is the shortfall that people will stumble on, that’s where people fall down. The housing makes all the difference; get the right housing and then things are much more likely to fall into place. Observation, site 2

The contextual challenges around housing were compounded by guidelines and restrictions on who could access certain social housing. Across both sites, there were restrictions on people leaving prison or those with particular criminal offences being able to access some private rented and social sector properties.

#### Time-limited and phased approach

The CTI model is typically implemented in three phases: transition to community, try out and transfer of care, each lasting 3 months. The CTI model delivered included a pre-CTI phase to build rapport and prepare service users for the transition from prison to community. The time-limited nature is a key focus of the intervention.

Fidelity to the time-limited and phased approach was routinely adhered to across sites, with the three-phase structure guiding the transition process for service users. CTI caseworkers explicitly outlined the distinct phases of the intervention and how this would guide their interactions. At both sites, adaptations to the level and intensity of support through the phases were observed, with more frequent meetings at the initial phase and less frequent check-ins once stability was achieved.

Despite this, instances were indicated where caseworkers expressed difficulty adhering to the strict time limits, especially when service users needed extended support or housing was not secured during the intervention period.

They could be referred to as other support, depending on where they are really and in their like position of accommodation. If they’ve just moved into accommodation, we refer to like tenancy support which is another 13 weeks then of support for their accommodation. But yeah, they know it’s for nine months. PID 105, caseworker, site 1

If we feel we need to, we can extend it … For instance, we’ve had [name] now for eight months I think before he got a tenancy … it just so happens that we found one, just before, so we’ll extend him now for another twelve weeks. Just to make sure he’s alright. PID 101, caseworker, site 2

The introduction of the ECSL and the Probation prioritisation framework^15 16^ resulted in the pre-CTI phase not being delivered as the CTI model intended. The pre-CTI phase is the initial stage of the intervention, where the focus is on caseworkers developing a trusting relationship with the service user. Conducted while the service user is still in custody, this phase allows caseworkers to build rapport, tailor support and ensure that essential needs, such as applying for benefits, are addressed. Without this groundwork, caseworkers reported that establishing trust and tailoring support became difficult, often leading to chaotic releases where vital needs, such as applying for benefits and securing benefits, went unmet. These contextual issues resulted in both sites not being able to deliver the pre-CTI phase as intended.

#### Caseloads and supervision

A key principle of the CTI model is small caseloads, team-based supervision and frequent case reviews. The training slides for the provision of the CTI intervention from the intervention delivery team indicated intended caseloads of 20 or fewer, monitored by a case management system. A meta-analysis identified that the average number of cases within a CTI caseload was 25.^17^ At site 1, there was only one prison providing referrals (at the time of the study) with a smaller capacity for providing referrals. This resulted in average caseloads for site 1 being 15 throughout the trial period. At site 2, CTI caseworkers reported higher caseloads, of between 18 and 28, having larger prisons in the area providing referrals. Discussions with CTI caseworkers at site 2 indicated that they felt that this was a low and manageable caseload, many having previously worked in the prison and probation system where they experienced significantly higher caseloads, suggesting some contextual factors in how ‘small caseloads’ were viewed. Additionally, caseloads were weighted as service users in the final phase required far less support than those in the early stages.

The role of the operational lead was crucial at both sites. Supervision was a key feature of how the sites managed their caseloads. Observations at each site witnessed how caseloads were managed in weekly group supervisory meetings, and the progress of each service user was reviewed considering any key events that might have happened. Additional one-to-one meetings with each member of the team and the CTI team lead also occurred.

I’ll also look at how that caseload is with [name] and talk through any particularly challenging cases, and hopefully um, you know, come up with a plan in terms of moving things forward if, you know, sometimes you might feel a bit stuck with some complexities, um so I do that. Um and then yeah, just try to work really um, I suppose collaboratively with [name], um making sure that, you know, I’m up to speed with as much as possible around what’s going on with CTI. PID 104, caseworker, site 1

Team cohesion and support are essential when working with this population. During one observation, a CTI caseworker was informed that a previous service user had committed suicide. This was upsetting to the caseworker, and the team rallied to support them. The complexities faced by a population of people leaving prison and experiencing homelessness are high. Sadly, the death of service users is often experienced by those supporting them. For this reason, staff support is essential to prevent burnout.

#### Person-centred approach/community focused

The person-centred aspect of CTI includes elements such as personalised goal setting, strengthening social support networks, providing flexible, needs-based support and empowering through skill-building.

Observations of the various phases identified collaborative goal setting between the CTI caseworker and service user. With service users indicating their priorities, such as employment, housing stability or social connections. This process was focused on empowering individuals to take ownership of their transition, with the CTI caseworker helping them establish practical, manageable steps towards these goals. The CTI caseworkers were observed assisting service users with both everyday and complex issues. Examples witnessed included getting access to a phone, setting up a bank account or with a general practitioner, and supporting them to engage with substance use services, alongside the focus on gaining housing.

CTI caseworkers would support service users in navigating complex systems, such as housing, healthcare or employment services, and advocating on their behalf when barriers arose. Examples observed included CTI caseworkers accompanying service users to appointments, assisting with completing paperwork (for bank accounts, applying for benefits and housing), or negotiating on their behalf if they encounter difficulties accessing services. This support helped prevent situations where individuals may have been overlooked, which can be common during times of transition.

A service user was living in their car and facing having their case closed by the local council due to part of owning a home with their partner. However, due to their conviction, they could not return to the property. The CTI caseworker was the sole spokesperson striving to have the local council provide accommodation, setting up meetings with various relevant groups to deal with the situation on the service user’s behalf. Observation, site 2

During early phases, the CTI caseworkers were observed teaching service users how to search for properties. They indicated the websites to use, and how to employ filters to identify suitable properties and would assist in drafting emails to send to potential property owners or management companies. Once viewings were set up, the CTI caseworkers would attend the properties with the service user when possible. If they could not attend, CTI caseworkers would provide some questions to ask about the property and, in some instances, an opportunity for the service user to call them during the viewing if they needed additional support. CTI caseworkers would often suggest attending with a family member if they could not attend.

Service user struggles with using technology and needs additional support. Currently at supported accommodation but required to leave within the month. The caseworker offers to connect with the team at the supported accommodation on the service user’s behalf to request they support the service user to use the computer at the residence and assist searching for properties. The service user agrees and the caseworker calls the supported accommodation team to set up. Observation, site 2

CTI caseworkers would help individuals connect or reconnect with family, friends or community groups. This could include coordinating family meetings, linking individuals with peer support groups, or assisting with finding community-based programmes that fit their interests and cultural backgrounds.

So, you’re, during that time, we should have explored the local area, to go like, I don’t know, there’s a community centre round the corner, or there’s your local GPs, and there’s your sports centre, or there’s a library there, or there’s a men’s group round the corner, there’s a walking group there. PID 101 site 2

### Harm reduction/recovery-orientated approach

CTI incorporates harm reduction and recovery principles, particularly as many service users can be involved in substance use recovery and experiencing mental health issues. Support should aim to connect with teams inside and outside of prison. In theory, this collaboration would ensure that support services are in place on release, facilitating a smoother transition between prison and community support.

Observations identified that during the referral process, potential service users were asked about their specific support needs regarding substance use recovery, physical and mental health, and any other areas of support they might require. It was explained that plans would be made to connect them with identified support services on release from prison.

CTI caseworkers strived to meet individuals’ housing needs, but the housing market sometimes resulted in service users being placed in accommodations unsuitable for their recovery needs. The lack of housing, especially in site 1, often leads to service users being placed in temporary accommodation. This situation necessitated an enhanced focus on harm reduction as the service user might be accommodated with other substance users, making it more difficult to maintain sobriety or reduce their use.

I think making it feel as if they are comfortable there and before that period, they’ve got somewhere to come to if they want to use anything that’s going on here. We’ve always got something going on, and that’s what[organisation] are really good at. There are always drop-in sessions; if they are having a bad day, they can come in for a coffee. But they’ve got that support there, and I think that’s what makes a difference. If they haven’t and they are spending all day every day in B&B, the likelihood is they’ve got an issue with substances, so they are going to start using again because they are surrounded by it in B&B as well or temporary accommodation. So, if they can start doing things that fill their days up, that does help, it works well for them. PID 105, caseworker, site 1

Examples of harm reduction included connecting service users with substance use recovery groups (attending with the service user in some cases), facilitating access to primary healthcare services for routine check-ups, and ensuring medication-assisted treatment continued postrelease, connecting service users to peer support groups, and providing immediate support and intervention for individuals experiencing mental health crises. Additional support provided included referring individuals to community resources for additional support, such as food banks and legal aid services. Site 2 had connections with local food stores and clothing brands, receiving food packages and clothing stock, which would be distributed among service users.

## Discussion

This study encountered several implementation challenges, notably limited recruitment and poor retention, which constrained the process evaluation and its ability to meet its intended aims. Despite these limitations, the data collected suggest that the intervention was well-received by caseworkers, who reported numerous success stories and perceived the model as acceptable and impactful. However, given their employment relationship with the intervention, there is a risk of positive bias in their assessments.

Observational data also indicated high levels of acceptability among service users, who valued the phased, person-centred support. However, the absence of direct interviews with trial participants limits the strength of these conclusions, as acceptability was inferred from continued engagement rather than explicitly reported experiences. The challenges experienced reflect those experienced in evaluating interventions with vulnerable populations, where ethical and logistical barriers often limit direct data collection.^18^

A key challenge was the individual-level randomisation, which was met with resistance from CTI teams who viewed it as ethically problematic to withhold a potentially beneficial intervention. This resistance was likely exacerbated by the fact that the intervention had already been implemented prior to the trial, and staff were already convinced of its value. This reflects broader findings in CTI research, where staff investment in the intervention can conflict with the principles of equipoise required in trial designs.^18^ The continued support from the charity’s executive team was crucial in maintaining trial integrity, highlighting the importance of organisational buy-in at all levels, particularly in settings with limited experience in research.^19^

The reluctance of staff to withhold services from control participants suggests that a cluster randomised design may be more appropriate in future trials to reduce contamination. This is especially relevant given that site 2 was delivering an alternative community-based model, which could confound outcomes and obscure the intervention’s true effect. In a full-scale trial, regional variations in service provision must be carefully accounted for, as areas with more robust support systems may reduce the observable impact of CTI for control participants. However, significantly powering such a study may be difficult.

Fidelity to the CTI model was generally maintained, with a strong emphasis on securing stable housing before addressing other needs. This aligns with the core principles of CTI, which prioritise housing as a foundation for reintegration.^9 10^ However, site 1 faced significant barriers due to a severe housing shortage, while site two benefited from stronger relationships with both social and private landlords. These disparities reflect broader structural issues in Wales, where access to social housing is constrained, particularly for single men leaving prison, who often face exclusion from social and private rented properties.^20^

The phased, time-limited nature of CTI was mostly adhered to, though caseworkers reported difficulties with strict timeframes. The pre-CTI phase, intended to begin in prison, was frequently missed due to contextual barriers, resulting in most support being delivered postrelease. This undermines a key strength of CTI: its ability to bridge institutional and community care. Nevertheless, caseworkers adapted flexibly, scaling support intensity based on individual needs and gradually reducing involvement over time.

The intervention’s person-centred approach emphasising personalised goal setting, social support and empowerment was evident in practice. Caseworkers supported service users with both everyday and complex challenges, helped them navigate fragmented systems and facilitated reconnections with family and community networks. These findings echo broader evidence that CTI can improve continuity of care, reduce homelessness and support reintegration for people leaving institutional settings.^9 18 19^

### Strengths and limitations

The study was limited by the trial design; we adopted standard recruitment and follow-up methods for general population research using clinical research nurses instead of tailoring this to the population of prison research. Data collection followed traditional transitory processes only engaging participants for specific elements, that is, consent and data collection. Whereas more successful studies in prison research have had teams of researchers experienced in prison research, who focused on building trust and rapport to reduce attrition while also conducting recruitment and data collection.^9 21^ A dedicated research team should have been formed who would be based at each intervention site throughout the project to be available for recruitment or follow-up at a moment’s notice.

Our findings are limited by the lack of trial participants available to be interviewed, with acceptability inferred from service users continuing to engage with the intervention. Those who found the model unacceptable likely did not engage with the CTI caseworkers and hence could not be observed. Additionally, the absence of perspectives from prison referrers, probation caseworkers and housing stakeholders limits our understanding of how acceptable the intervention is to those responsible for making referrals.

Regardless of the challenges faced, piloting a randomised trial within a prison setting is an achievement, given the logistical and ethical challenges involved, and this pilot highlights how formidable these are under the current overcrowding issues within prisons. The key strengths of this evaluation are our understanding of fidelity of the intervention and the learning around pitfalls to avoid around individual level randomisation of a pre-existing intervention.

## Conclusions

This study faced significant challenges which impacted its aims. Despite these issues, the evaluation provided valuable insights into conducting trials on interventions for people leaving prison. The challenges experienced should be learnt from and lead to improved study designs for future work. The population of people leaving prison at risk of homelessness are some of the most vulnerable in our society, and it is important that we try to support them if we want to reduce recidivism, health burdens and inequity within society.

## Supplementary material

10.1136/bmjopen-2024-097761 online supplemental file 1

## Data Availability

Data are available on reasonable request.
