# Supplementary material for: A pilot randomised controlled trial of a critical time intervention for people leaving prison: findings from an integrated process evaluation
Source: BMJ Open. 2025 Dec 17;15(12):e097761. doi: 10.1136/bmjopen-2024-097761 (PMC12716555; doi:10.1136/bmjopen-2024-097761)

PHaCT Protocol

Preventing Homelessness, improving health for people leaving prison: a pilot randomised controlled trial of a Critical Time Intervention

V2.2 11.04.2024

**Sponsor:**	University of Glasgow<br>University Avenue, Glasgow G12 8QQ
**Sponsor ref:**	N/P
**Funder:**	NIHR
**Funder ref:**	NIHR134281
**REC ref:**	22/WA/0347
**IRAS number:**	319937
**ISRCTN ref:**	46969988
**Q-Pulse Document Template Number:**	TPL/003/2

SIGNATURE PAGE

The undersigned confirm that the following protocol has been agreed and accepted and that the Chief Investigator agrees to conduct the trial in compliance with the approved protocol and will adhere to the principles outlined in the relevant trial regulations, GCP guidelines, and CTR's SOPs.

I agree to ensure that the confidential information contained in this document will not be used for any other purpose other than the evaluation or conduct of the clinical investigation without the prior written consent of the Sponsor.

I also confirm that I will make the findings of the trial publicly available through publication or other dissemination tools without any unnecessary delay and that an honest accurate and transparent account of the trial will be given; and that any discrepancies from the trial as planned in this protocol will be explained.

**Trial sponsor:**			
[redacted]	[redacted]	[redacted]	14.05.2024
Name	*Position*	*Signature*	*Date*
**Director:**			
[redacted]	[redacted]	[redacted]	15.04.2024
Name	*Position*	*signature*	*Date*
**Chief Investigator:**			
[redacted]	[redacted]	[redacted]	14.04.2024
Name	*Position*	*Signature*	*Date*

General Information This protocol describes the PHaCT clinical trial and provides information about the procedures for entering participants into the trial. The protocol should not be used as a guide, or as an aide-

memoire for the treatment of other participants. Every care has been taken in drafting this protocol; however, corrections or amendments may be necessary. These will be circulated to the known Investigators in the trial. Problems relating to the trial should be referred, in the first instance, to CTR.

Contact details – Chief Investigator / Co-Investigators

CHIEF INVESTIGATOR

**Title and name**	**Professor Jim Lewsey**
**Position**	[redacted]
**Address**	[redacted]
**Postcode**	[redacted]
**Tel**	[redacted]
**E-mail**	[redacted]

CO-INVESTIGATORS

[redacted]	[redacted]
[redacted]	[redacted]
[redacted]	[redacted]
[redacted]	[redacted]
[redacted]	[redacted]

Trial Co-ordination:
The PHaCT trial is being coordinated by the Centre for Trials Research (CTR), Cardiff University, a Clinical Research Collaboration (UKCRC) registered trials unit.

This protocol has been developed by the PHaCT Trial Management Group (TMG).

For **all queries** please contact the PHaCT team through the main trial email address. Any clinical queries will be directed through the Trial Manager to either the Chief Investigator or a Co-Investigators

**Main Trial Email:**	PHaCT@Cardiff.ac.uk	
Trial Administrator:	[redacted]	[redacted]
Senior Trial Manager:	[redacted]	[redacted]
Trial Manager	[redacted]	[redacted]
Data Manager:	[redacted]	[redacted]
Trial Statistician:	[redacted]	[redacted]
Qualitative Researcher	[redacted]	[redacted]
Senior Health Economist	[redacted]	[redacted]
Health Economist	[redacted]	[redacted]
CTR Director:	[redacted]	[redacted]

Queries:

Queries

PHaCT@Cardiff.ac.uk

All queries will be directed to the most appropriate person.

Safeguarding concerns:

Safeguarding reporting

Any safeguarding concerns should be reported within 24 hours of becoming aware of them either by:

1. Contacting the trial administrator on 02922 514511 or PHaCT@cardiff.ac.uk using only the Participants PID number.

Contents

SIGNATURE PAGE 1

Contact details – Chief Investigator / Co-Investigators 2

1. Amendment History 8

2. Synopsis 9

3 Trial summary & schema 11

3.1 Full study schema (v2 17.07.2023) 11

3.2 RCT Participant flow diagram (v1.1 17.10.2022) 12

3.3 Trial lay summary 13

4 Background 14

4.1 Rationale for current pilot trial 17

5 Trial objectives/endpoints and outcome measures 17

5.1 Primary outcomes measure(s) 18

5.2 Secondary outcomes measures 18

6 Trial design and setting 19

6.1 Risk assessment 19

7 Site and Investigator selection 20

8 Participant selection 20

8.1 RCT and non-RCT participants Inclusion criteria 20

8.2 RCT Exclusion criteria 20

9. Recruitment, Screening, and Registration 20

9.1 RCT PARTICIPANTS 20

9.2 NON-RCT PARTICIPANTS 22

10. Withdrawal & lost to follow-up 23

10.1 RCT Withdrawal 23

10.2 RCT Strategies to minimise loss to follow up 23

10.3 NON-RCT Withdrawal 23

11. Trial Intervention 24

11.1 Critical Time Intervention 24

11.2 Compliance 25

11.3 Training 25

12 Trial procedures 25

12.1 Assessments 29

Figure 1. Schedule of enrolment, interventions and assessments 29

12.2 Follow-up 30

13 Safety reporting 31

13.1 Definitions 32

13.2 Reporting process 32

14 Statistical considerations 32

14.1 Randomisation 32

14.2 Blinding 33

14.3 Sample size 33

14.4 Missing, unused & spurious data 33

14.5 Procedures for reporting deviation(s) from the original SAP 33

14.6 Termination of the trial 33

15 Analysis 33

15.1 Main analysis 33

15.2 Qualitative analysis 34

15.3 Economic evaluation 35

15.4 Routine data analysis 36

16 Data Management 37

16.1 Data collection 39

stor16.2 Completion of CRFs 39

17 Protocol/GCP non-compliance 40

18 End of Trial definition 40

19 Archiving 40

20 Regulatory Considerations 40
20.1 Ethical and governance approval 40
20.2 Data Protection 41
20.3 Indemnity 41
20.4 Trial sponsorship 41
20.5 Funding 41
21 Trial management 41
21.1 TMG (Trial Management Group) 41
21.2 TSC (Trial Steering Committee) 42
22 Quality Control and Assurance 42
22.1 Monitoring 42
22.2 Audits & inspections 42
23 Publication policy 42
24 Milestones 43
25 References 43
26 Appendices 49
26.1 Logic model 49

Glossary of Abbreviations

**AUDIT-C**	Alcohol Use Disorders Identification Test-Consumtion
**CI**	Confidence interval
**CONSORT**	Consolidated Standards of Reporting Trials
**CRF**	Case Report Form
**CRN**	Clinical Research Network
**CTI**	Critical Time intervention
**CTR**	Centre for Trials Research
**DMC**	Data Monitoring Committee
**DMP**	Data Management Plan
**EoI**	Expression of interest
**EQ-5D**	EuroQoL standardised measure of health-related quality of life
**GCP**	Good Clinical Practice
**HMP**	Her Majesty's Prison
**ICECAP-A**	ICEpop CAPability measure for Adults
**ICH**	International Conference on Harmonization
**IQR**	Interquartile Ranges
**ISRCTN**	International Standard Randomised Controlled Trial Number
**MoJ**	Ministry of Justice
**MRC**	Medical Research Council
**NICE**	National Institute for Health and Care Excellence
**NIHR**	National Institute for Health and Care Research
**NHS**	National Health Service
**NW CRN**	Northwest Clinical Research Network
**PID**	Participant Identification Number
**PPI**	Patient and Public Involvement
**QA**	Quality Assurance
**QALY**	Quality-adjusted Life Years
**QAP**	Qualitative data collection and Analysis Plan
**QC**	Quality control
**QL (QoL)**	Quality of Life
**R&D**	Research and Development
**RCT**	Randomised Controlled Trial
**REC**	Research Ethics Committee
**SAIL**	Secure Anonymised Information Linkage
**SAP**	Statistical Analysis Plan
**SD**	Standard Deviation
**SF-12**	12-item Short Form health survey
**SOP**	Standard Operating Procedure
**TMF**	Trial Master File
**TMG**	Trial Management Group
**TSC**	Trial Steering Committee
**UK**	United Kingdom
**VOI**	Value of Information

1. Amendment History

The following amendments and/or administrative changes have been made to this protocol since the implementation of the first approved version.

Amendment No.	Protocol version no.	Date issued	Summary of changes made since previous version
3	2.2	11.04.2024	1. Change to progression criteria, removal of 12-month specification.<br>2. Change to end date.
2	2.1	04.01.2024	3. Added 5th potential site, [redacted]<br>4. General edits to language.
1	2.0	11.07.2023	1. Updated team members details<br>2. Updated references to 'NHS Digital' to 'NHS England' in line with recent name change<br>3. Inclusion of non-RCT participant interviews and observations to expand recruitment pool.<br>4. Broadening of recruiting team from 'R&D/CRN' to 'recruiter' to enable Cardiff trial team study staff to support recruitment in local sites.<br>5. Added ability to book follow-up visit appointment for baseline selection following TSC advice due to short prison slots.<br>6. Updated Logic model to map against key research questions

2. Synopsis

**Short title**	Preventing Homelessness, improving health for people leaving prison: a pilot randomised controlled trial of a Critical Time intervention
**Acronym**	PHaCT
**Internal ref. no.**	1095
**Development phase**	Phase II
**Funder and ref.**	NIHR134281
**Trial design**	Pilot Randomised controlled trial
**Trial participants**	Men at risk of homelessness leaving prison
**Planned sample size**	80 (1:1 randomisation intervention and usual care) allowing for 40% drop out at final follow-up
**Planned number of sites**	5 prisons ([redacted])
**Inclusion criteria**	1. Aged over 18 years.<br>2. Released into the [redacted] local authority areas and have a local connection (e.g., lived their previously; close relatives living in these areas)<br>3. Recourse to public funds<br>4. Have experienced prison and homelessness at least once.<br>5. Mental health or substance use support needs
**Exclusion criteria**	1. Individuals under the Multi-Agency Public Protection arrangements 3 (MAPPA 3)<br>2. Those benefitting from Housing First
**Treatment duration**	9 months consisting of 3 phases lasting 3 months each ('Transition to the Community', 'Try out', 'Transfer of Care')
**Follow-up duration**	3-, 6-, 9- and 12-months post randomisation
**Planned trial period**	33 months
**Primary objective**	To conduct a pilot RCT of housing led CTI for males leaving prison at risk of homelessness to determine if a full scale RCT is warranted.
**Secondary objectives**	1. To identify suitable and acceptable trial methods (recruitment, randomisation, intervention delivery, retention).<br>2. To identify feasible data collection methods and acceptable outcome measures.<br>3. To explore potential for data linkage in this population (acceptability in terms of consent, suitable data sets).

**Primary outcomes**	Meeting progression criteria to full scale trial:<br>1. Recruitment of 50% of those approached<br>2. Retention of 60% of those enrolled at post randomisation<br>3. Acceptability of intervention delivery to participants and staff 4. Intervention delivered with fidelity
**Secondary outcomes**	1. Health-related quality of life (SF-12, (total, physical and mental health scores); EQ-5D; ICECAP-A)<br>2. Housing stability (i.e., number of days in a stable accommodation)<br>3. Substance use and hazardous alcohol consumption<br>4. Deaths (including cause of death)
**Tertiary/Exploratory outcomes**	We will investigate the possibility of gaining data on:<br>1. Re-incarceration<br>2. Support networks<br>3. Crime (e.g., criminal arrest, criminal convictions, police contacts, rearrest)<br>4. Education training and employment<br>5. Finance, benefits and debt<br>6. Support network/family & friend connections<br>7. Wellbeing<br>8. Community integration and engagement
**Intervention**	Critical Time Intervention (CTI) delivered by charity case workers to improve an individual's engagement with treatment and community services through developing problem-solving skills and are targeted to continuity of care during a period of transition (i.e., release from prison)

3 Trial summary & schema

3.1 Full study schema (v2 17.07.2023)

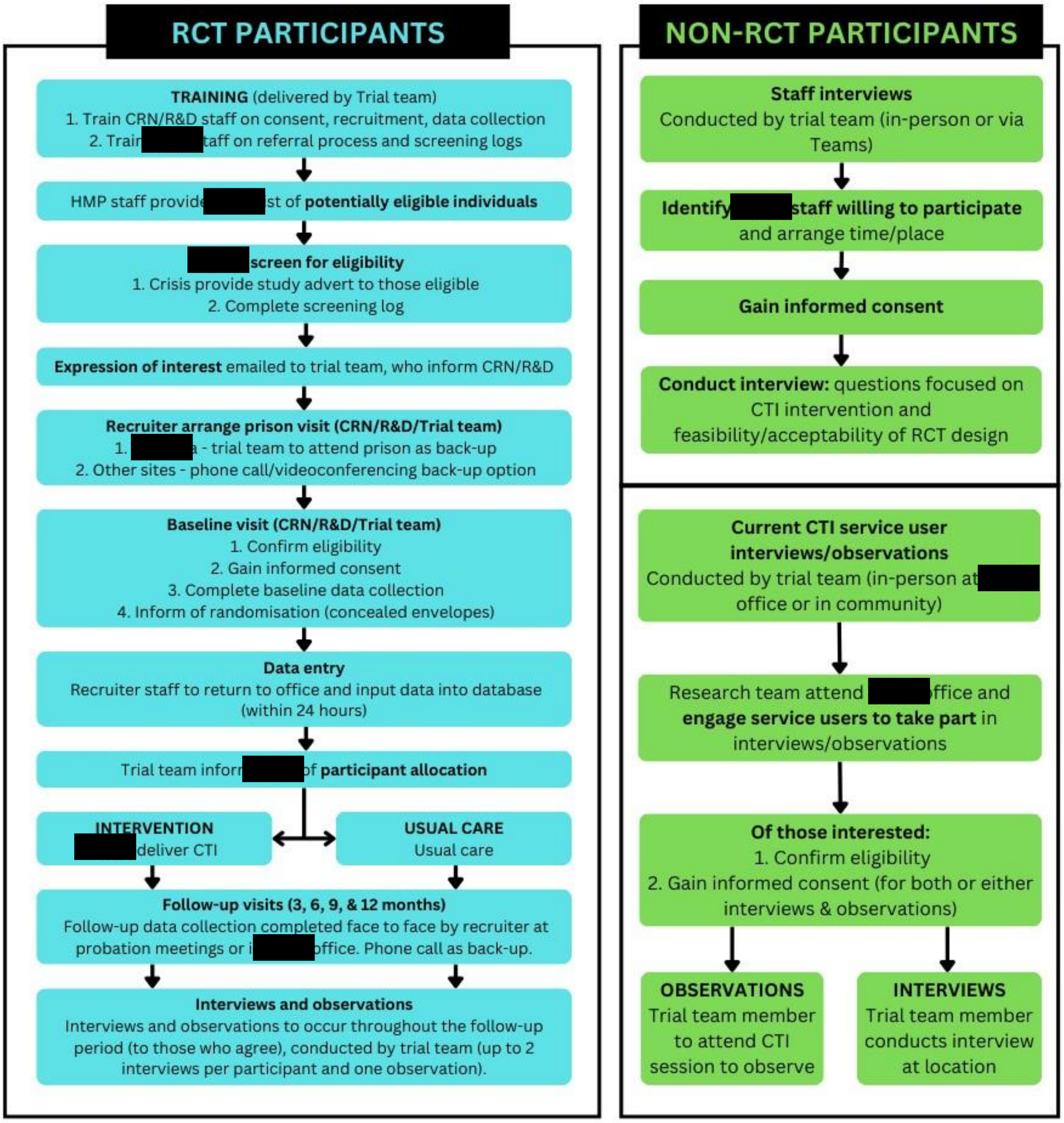

3.2 RCT Participant flow diagram (v1.1 17.10.2022)

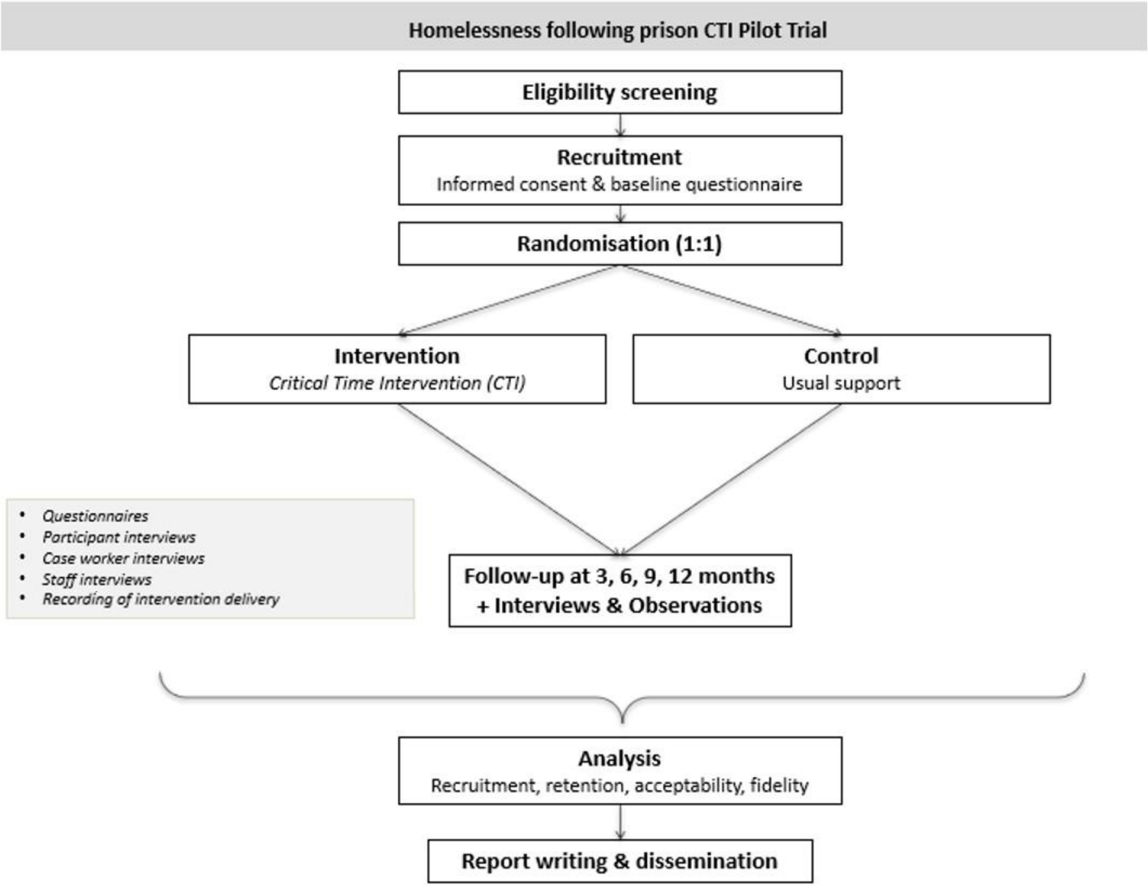

3.3 Trial lay summary

People who have been in prison and are released back into the community are at a high risk of homelessness. Nearly 1 in 2 who are released do not have a settled home and 1 in 3 are homeless or are in unsettled housing one year later. The life expectancy of people without a stable home is much lower and they have a worse quality of life. There are also wider society impacts of homelessness such as lower community wellbeing, more crime and higher healthcare and justice costs. Crisis (a national charity for people experiencing homelessness) have calculated that on average the yearly cost of one person rough sleeping could cost £20,128. Although there are good interventions to stop homelessness, they are mostly from the US, and we do not know how these work for people who have been in prison. Critical Time Intervention (CTI) is the name of a type of intervention which aims to support those most in need in society during times of important life changes. They help people at risk of homelessness stabilise their housing situation, particularly those people who are leaving institutions (like prisons). Homelessness services think that this type of intervention works best when it is 'housing-led'. This is where people are given housing quickly without conditions. There have been no studies which have looked at how good housing led CTIs are at stopping homelessness and better the health of people with prison experience in the UK and if they are good value for money.

Working with a charity we will carry out a 'pilot Randomised Controlled Trial (RCT)' (where people are randomly allocated to one of two groups: CTI or usual care) in four prison populations in England and Wales. In the long term we plan to test if a housing-led CTI betters people's health-related quality of life and housing ('effectiveness') and is 'cost-effective' (i.e., its good value for money), but first we need to find out if the intervention and study design (research methods) are acceptable.

We will recruit 80 adult males who are ready to leave one of 4 prisons ([redacted]) who are at risk of homelessness to take part. Participants will be asked to complete a survey asking about their health-related quality of life, housing stability, substance misuse, alcohol consumption, re-incarceration, support networks etc. We will also ask their permission to access anonymous routinely collected health, educational and aggregated criminal justice information. Participants will then be randomly allocated to either get the CTI or usual care. We will follow-up participants at 3, 6, 9 and 12 months. Some participants, prison staff and intervention staff will also be asked to take part in face-to-face interviews to find out more about their experiences of taking part to find out if they think the intervention and recruitment methods are feasible and acceptable to them. Some of their sessions with their CTI worker will also be observed to see how the CTI intervention is being delivered.

4 Background

For people leaving prison, re-integrating into the community can be a difficult process. Substance use, poor mental health, personal and situational factors such as low levels of literacy and absence of a stable support network [1], reinforced by a lack of affordable housing and employment opportunities, make them particularly at risk of homelessness. The absence of stable accommodation has a devastating impact on physical and mental health – people experiencing homelessness are more exposed than the general population to infectious and noncommunicable diseases [2], mental health problems (including alcohol and substance use) [3], have higher rates of emergency hospital admissions [4] and report lower levels of wellbeing and health-related quality of life [5, 6]. Poor nutrition, inadequate self-management of preventable conditions and poor adherence to medications contribute to an all-cause mortality rate which is between 3 and 11 times higher in people experiencing homelessness, compared to other socio-economically disadvantaged people [7, 8]. Further, absence of stable accommodation is also strongly associated with unemployment [9] and criminal activity [10, 11]. Homelessness also has a broader societal impact; in terms of decreased community wellbeing (e.g., perceived safety) and higher costs (e.g., increased utilisation of judicial services and welfare sectors) [12]. An analysis conducted by Crisis, a national charity for people experiencing homelessness, has estimated the average annual cost associated with a single case of street homelessness could reach £20,128 [13]. In consideration of its detrimental health and societal consequences, homelessness is a major public health issue which demands an effective preventive approach. This will generate substantial benefits in terms of housing stability, health and wellbeing, and wider societal cost savings.

A UK survey conducted with nearly 500 single people experiencing homelessness across 16 local authorities found that 41% of the interviewees had once served a prison sentence [14]. Although the need to provide housing assistance to those leaving prison has been recognised in homelessness and offender policy and legislation across the UK administrations (e.g., the Offender Rehabilitation Act (2014) [15], the Housing (Wales) Act (2014) [16], the Homelessness Reduction Act (2017) [17]), to date, efforts to meet the housing needs of people on release from prison have not proven effective [18], nor public health interventions routinely implemented to support people in prisons during the critical transition to the community.

Critical Time Interventions (CTIs) are focused and time-limited case management interventions that aim to improve an individual's engagement with treatment and community services through developing problem-solving skills and are targeted to continuity of care during a period of transition (e.g., release from prison) [19]. The key elements of CTIs are small caseloads, active community

outreach, individualised case management plans, psychosocial skill building, motivational coaching, with a phased approach that decreases in intensity over time [20]. CTIs, by providing emotional and practical support during critical times, endeavour to strengthen long-term ties to services as well as family and friends [21].

A CTI was first developed and evaluated for people with mental illness who were transitioning from shelters to housing in the community [22] and has been adapted and used in other settings (e.g., after discharge from psychiatric hospitalisation [23], for families experiencing homelessness and mental health problems [24] and housing-unstable families with children in foster care [25]).

A key concern for people leaving prison is access to suitable accommodation upon release. In a nested qualitative study as part of a randomised controlled trial (RCT) of CTI for people in prison with severe mental illness [26], health professionals stated that securing suitable accommodation was arguably the top priority for people newly released from prison because it provides a stable base from which to address other re-integration into the community concerns. This was echoed in the views of the RCT participants who stated that finding suitable accommodation was vital for accessing key services and reducing their risk of future reoffending and incarceration [27]. In a mixed-methods study examining the needs and obstacles people who had been in prison face upon release [28], most participants identified housing as crucial in promoting successful re-integration into the community. These findings are consistent with 'Housing First' interventions (assisting people with access to permanent housing as the key initial step in addressing homelessness) being successful in improving housing stability and some aspects of health [29].

Existing evidence:

In a systematic review of case management for people experiencing homelessness [30], two CTI studies were identified – one RCT [31] and one historically controlled study [23]. In the RCT, both the CTI and control groups (usual services only) were discharged from an on-site psychiatry program in a shelter to community-based housing in the New York City region. The CTI group reported significantly fewer homeless nights and a decrease in negative psychiatric symptoms. In the historically controlled study, set in eight sites in the US, housing was not provided as part of the CTI. Those receiving CTI spent more days housed, fewer days in institutional settings, lower alcohol/drug use and less psychiatric problems than those receiving usual services.

In a more recent systematic review of case management for people experiencing homelessness or people who are vulnerably housed [19], five CTI RCT studies were identified – one identified in the

previous review [31], with the others being set in the Netherlands [32, 33] and the New York region [21, 24, 34]. Of the four RCTs that had housing stability outcomes, one found no significant improvement in outcome for the CTI group [32], two found significant reductions in odds of homelessness [21, 31] and one found higher rates of time spent in housing for the CTI group, but findings converged when CTI services ended [24]. All studies (or in follow-up research) had mental health outcomes; two finding no significant improvement in outcomes [32, 33], one showing a reduction in internalising and externalising behaviours in children under 5 years [24] but no effect on maternal mental health [34], one finding decreases in negative psychiatric symptoms [31] and one finding decreases in psychiatric re-hospitalisation [35]. Only two studies had quality of life as an outcome of interest and both reported no differences between CTI and control group [32, 33].

From our own literature searches using key terms in the aforementioned reviews, and from citation tracking of the key studies above, we found two further CTI RCTs [26, 36], a before and after study [37], a cost-effectiveness study (from a RCT) [38] and a rapid evidence assessment of homelessness services [39]. The effectiveness studies found that those in CTI group had greater continuity of psychiatric outpatient care [36], increased engagement with psychiatric services at six months (but not observed at one year follow-up) [26] and reduced homelessness and psychiatric symptoms [37]. In a cost-effectiveness study from the US, the CTI was found to be cost-effective compared to the status quo for people experiencing homelessness with severe mental illness [38]. The rapid evidence assessment concluded that CTIs were effective services and, more generally, homelessness services that incorporate permanent supported housing elements are effective. The review authors identify RCTs across multiple sites and robust economic evidence (especially in the UK) as gaps in the evidence base.

In summary, there exists some high-quality evidence that CTI improves housing stability outcomes in people experiencing homelessness or groups that are vulnerably housed. The results from the same evidence base on mental health outcomes is not as strong but the majority of studies found CTI achieving favourable outcomes. Only two studies had quality of life as an outcome and only one study, from the UK, was based in a prison. That study [26] did not provide housing as part of CTI, yet provision of suitable accommodation is seen as vital for promoting successful re-integration into the community. In fact, there were only two studies, both from the US [24, 37], that provided housing as part of a CTI intervention. While those two studies suggested the intervention may be effective in improving health, the nature of the housing market and social security support provided in the UK differ markedly and therefore limit generalisability of that evidence.

4.1 Rationale for current pilot trial

The adverse health impacts of homelessness are compounded by the experience of imprisonment, with health outcomes substantially worse amongst people with a history of both imprisonment and homelessness than either in isolation [40]. In collaboration with [redacted] charity for single people experiencing homelessness ([redacted]), we will pilot a RCT of a housing-led CTI in four prisons, located in Wales and England. The quantitative and qualitative evidence generated will inform whether a future definitive full-scale trial of the CTI is warranted.

5 Trial objectives/endpoints and outcome measures

The overall aim is to conduct a pilot RCT in four male prisons to determine whether a future fullscale RCT assessing effectiveness and cost-effectiveness of a housing-led CTI is warranted. The research questions are:

1 **Intervention and randomised trial delivery**

1.1 Is the intervention acceptable to participants, those delivering and those funding?

1.2 What do qualitative and quantitative data suggest in terms of refinements to programme theory, implementation, fidelity, reach, scalability, and acceptability?

1.3 Are there potential harms and unintended consequences of the intervention? How might these be reduced? How can these be measured?

1.4 Are there any potential ethical, practical, statutory, or other legal barriers in running a trial in this population?

2 **Recruitment**

2.1 What proportion of eligible people in prison are recruited?

2.2 What proportion of recruited participants are retained?

2.3 What proportion of people in prison provide consent for linkage to routine health and criminal justice data?

3 **Data collection procedures**

3.1 Are methods of data collection feasible and what refinements (if any) are needed?

3.2 Are outcome measures suitable and what refinements (if any) are needed?

3.3 What consent and governance requirements are acceptable to data providers for linkage to routine health, welfare, and criminal justice data?

3.4 What are the likely sources of data on the health and broader social impacts of the intervention to inform an economic evaluation in a full-scale trial?

5.1 Primary outcomes measure(s)

The primary outcome is progression criteria that will determine whether a full-scale RCT of the intervention is warranted. The progression criteria are:

1) Recruitment: 50% of those approached agree to participate.
2) Retention: 60% of those recruited, in both intervention and control groups, are retained at final follow-up.
3) The process evaluation provides evidence that the process is acceptable for participants and staff delivering the intervention.
4) The intervention is implemented with fidelity in all settings.

The progression criteria will be agreed in collaboration with an independent Trial Steering Committee (TSC), that will carefully consider progress against these criteria using thresholds for progression according to a traffic light system (green: all criteria are met, the study should progress as designed in this pilot; amber: the majority of criteria are met and with adaptations to methods all criteria could be met; red: the minority of criteria are met and the study should not proceed).

5.2 Secondary outcomes measures

Indicative outcome measures for a full-scale trial will be evaluated and will include:

1. **Primary:** Health-related quality of life (SF-12 total score)
2. **Secondary:**
   - Health Related Quality of Life (EQ-5D)
   - Housing stability (i.e., number of days in a stable accommodation)
   - Substance use and hazardous alcohol consumption.
   - Deaths (including cause of death and hospital deaths)
   - Capability wellbeing (ICECAP-A)
3. **Exploratory:**
   - Percentage of participants who consent to link data their data anonymously with routine electronic data through SAIL, NHS England and Ministry of Justice Data Lab.

- Percentage (of those who consented) of successfully linked records with data held in SAIL, NHS England and Ministry of Justice Data Lab to further inform additional secondary outcomes, for example: ○ Re-incarceration ○ Support networks
  - Crime (e.g., criminal arrest, criminal convictions, police contacts, rearrest) ○ Education training and employment ○ Finance, benefits, and debt
  - Support network/family & friend connections ○ Wellbeing
  - Community integration and engagement

6 Trial design and setting

This is a multicentre phase 2 pilot Randomised Control Trial (RCT) with an integrated process and economic evaluation. Eighty men leaving prison at risk of homelessness will be recruited and randomised on a 1:1 ratio (stratified by prison) to take part in a CTI or receive usual care. Sites include five male prisons ([redacted]) where housing-led CTI has recently been introduced and implemented in the first half of 2021. Participants will complete a survey at baseline and followed up at 3-, 6-, 9- and 12-months post randomisation. A subsample of intervention participants (n=12 (x 2 interviews)), control participants (n=16), prison staff (n=6) and interventionists (n=6) will be sampled to maximise variability (based on age, prison, caseworker, differing levels of engagement with the trial (and the intervention specifically), and phases of delivery) to take part in repeated (intervention participants only)/one-off interviews. We will additionally recruit n=12 individuals currently taking part in CTI intervention but not recruited on the trial to take part in a one-to-one interview to find out more about their experiences of the inte4rventions and thoughts on the proposed recruitment methods. Consent will be sought to link with participants routinely collected electronic health records via SAIL and NHS England as well as augmented Criminal Justice data via the Ministry of Justice DataLab.

6.1 Risk assessment

A trial Risk Assessment has been completed to identify the potential hazards/risks associated with the trial and to assess the likelihood of those hazards/risks occurring and resulting in harm. This risk assessment will include:

- The known and potential risks and benefits to human participants
- How high the risk is compared to normal standard practice.
- How the risk will be minimised/managed

This trial has been categorised as a low-risk trial, where the level of risk is comparable to the risk of standard care. A copy of the trial risk assessment may be requested from the Trial Manager.

7 Site and Investigator selection

This trial will be carried out at five male prisons in England and Wales ([redacted]) where CTI has recently been introduced and is being delivered by CTI workers employed by the charity delivering the intervention.

8 Participant selection

Participants are eligible for the trial if they meet all of the following inclusion criteria and none of the exclusion criteria apply. All queries about participant eligibility should be directed to the Trial Manager before randomisation/registration. Participants are split between those involved in the RCT and those currently receiving CTI who will only be interviewed/observed.

8.1 RCT and non-RCT participants Inclusion criteria

Men leaving prison at risk of homelessness can be included if they are:

1. Aged over 18 years.
2. Released into the [redacted] local authority areas and have a local connection (e.g., lived there previously; close relatives living in these areas)
3. Recourse to public funds
4. Have experienced prison and homelessness at least once.
5. Have mental health or substance use support needs.

8.2 RCT Exclusion criteria

Men leaving prison cannot take part if they are:

1. under the Multi-Agency Public Protection panel 3 (MAPPA 3)
2. in receipt of Housing First (i.e., support needs too high/complex to benefit from CTI)

9. Recruitment, Screening, and Registration

9.1 RCT PARTICIPANTS

9.1.1 Participant identification:

Potential participants will be referred to the CTI intervention team for eligibility screening by the Community Offender Management Team or Probation service. The CTI intervention team will screen

potential participants at weekly allocation meetings to see if an individual meets the trial inclusion/exclusion criteria and will invite eligible individuals to take part in the research (using the expression of interest form which provides a summary of the study). Where participants express an interest in taking part, CTI staff will pass on their details to the research team using an Expression of Interest (EoI) from. Following this, the recruiter ([redacted] PHaCT trial team) will arrange access to the prison for the baseline visit with support of the local CTI staff using the prison visitor scheme.

9.1.2 Screening logs:

will be kept by CTI staff for each site to record all referrals received regardless of eligibility status and whether they were approached/consented or not. Screening logs will capture non-identifiable information on a tally system relating to referral route, eligibility status, EoI, recruitment status, reasons for non-eligibility or non-recruitment (where possible). Logs will be completed electronically by individually designated CTI staff for each site. Where there are multiple staff recruiting at one site, entries will be aggregated onto one log. Data entered onto the log will only be accessible to named trial staff members which will be outlined in the Site Delegation Log. Logs will be returned to the Data Manager on a weekly basis using the trial email address.

9.1.3 Recruitment:

A total of 80 participants will be recruited. We will also recruit a sub-sample of intervention participants (n=12 (x 2 interviews)), control participants (n=16) prison staff and interventionists (n=12) to take part in face-to-face repeated (intervention participants only)/one-off interviews.

9.1.4 Informed consent:

During the baseline visit the potential recruit will be provided with a participant information sheet and trained recruiter will go over the details explaining the risk and benefits of taking part to the potential participant. It will be explained to them that all information they provide, including their personal details, will be kept strictly confidential and will only be accessible to the immediate research team at Cardiff University and that they have the right to withdraw from the study at any time without having to give a reason. Potential participants will be provided with sufficient time to consider their participation and will have multiple opportunities to ask any questions. We might have to share personal data with the University of Glasgow as the Sponsor of this study so that they can check we are following legal processes. This will be managed in strict confidence.

Should they agree to take part, written informed consent will only be obtained by the recruiter (who will be trained in taking informed consent) using the trial Consent Form. A Site Delegation Log will outline who is responsible for taking participants consent and will be kept centrally with the trial team. Participants will be asked to sign two copies (one copy to be kept by the participant and the other to be returned to the TM via registered mail). Only once written informed consent has been obtained from the participant will they be considered a trial participant.

Participants will be asked to provide written consent to take part in a potential qualitative interview, although it will be explained to them that they may not be sampled for this aspect of the trial. It will be further explained that if they later choose not to take part in this aspect of the trial, that this will not impact on their valuable contribution made to the trial so far. They will also be asked if they are willing to consent to trial staff requesting access to their anonymised electronic routine data through a third-party data provider. Participants consent to take part will be verbally reaffirmed at each data collection point.

9.1.5 Randomisation:

Participants will be randomised to receive CTI or usual care following gaining informed consent and completion of the baseline assessment by the recruiter. Randomisation will be on a 1:1 ratio and stratified by prison, using random blocks, and generated by the trial statistician and checked by the senior statistician. Allocation will be revealed to participants by the recruiter by opening a sequential sealed envelope. and communicated to the participant by the recruiter (using a prepared script). Owing to the nature of the intervention, it will not be possible to blind participants, recruiter or CTI staff to the treatment allocation.

9.2 NON-RCT PARTICIPANTS

9.2.1 Participation identification:

Those currently receiving CTI from the charity will be invited to take part in an interview and allow observations of their meetings (maximum 3 per person).

9.2.2 Recruitment:

A maximum of 12 interviews and observations will be conducted.

9.2.3 Informed consent:

Those indicating interest will be provided a participant information sheet and PHaCT research staff will go over the details explaining the risk and benefits of taking part to the potential participant. All details of the PIS and CF will be explained with opportunities to ask any questions. Paper consent forms will

be used. Participants will be asked to sign two copies (one copy to be kept by the participant and the other to be returned to Cardiff University for storage). Only once written informed consent has been obtained from the participant will they be interviewed/observed.

10. Withdrawal & lost to follow-up

10.1 RCT Withdrawal

During recruitment it will be explained to potential participants that they have the right to withdraw consent for participation in any aspect of the trial without having to provide a reason. Participants care will not be affected at any time by declining to participate or withdraw from the trial. However, whenever a reason is given, this will be recorded by the study team on a study withdrawal form following standard CTR processes and used anonymously for the process evaluation.

If a participant initially consents but subsequently withdraws from the trial, clear distinction must be made as to what aspect of the trial the participant is withdrawing from (i.e., partial withdrawal from questionnaire interview etc. withdrawal of all data collection, withdrawal from intervention etc.) and recorded on a withdrawal form.

10.2 RCT Strategies to minimise loss to follow up

We aim to follow-up all participants recruited into the trial. To maximise our follow-up success rate where possible we will aim to follow up participants in person and brokered via the participants probation officer. Where it's not possible to follow-up in person, we will attempt to contact participants via alternative contact details they have provided us, or updated details provided by the probation officer. We will make 4 attempts to follow-up with participant at each timepoint. Where a participant has been lost to follow-up at one time point, they will still be contacted at the next followup timepoint, unless they have explicitly withdrawn from follow data collection. Information regarding follow-up routes and contact attempts will be collected for the process evaluation.

10.3 NON-RCT Withdrawal

During recruitment it will be explained to potential participants that they have the right to withdraw consent for participation in any aspect of the trial without having to provide a reason. Participants' care will not be affected at any time by declining to participate or withdraw. However, whenever a reason is given, this will be recorded by the study team on a study withdrawal form following standard CTR processes and used anonymously for the process evaluation. If a participant initially consents but subsequently withdraws from the trial, we will inquire if they wish for all previously recorded data to be removed and act accordingly.

11. Trial Intervention

11.1 Critical Time Intervention

The Critical time Intervention (CTI) was first developed by mental health clinicians and researchers working in New York City men's shelters in the 1980s and 1990s [41]. It was noted that despite clear discharge planning, a large proportion of men experienced homelessness within 18 months of discharge from shelters [42]. The CTI approach is a psychosocial individual-level intervention that facilitates integration and continuity of care during a time of transition from institution to community settings [43]. It achieves this by ensuring that an individual has long term ties to services, family and friends through providing time-limited, but direct, emotional and practical support during the transition. CTI participants create a personal development plan and identify short-medium-long term goals depending on their individual needs, with the aim to develop self-management skills, feeling of empowerment and improving their health and quality of life. Housing provision provides additional stability, removing practical and emotional issues tied to experiencing homelessness.

Key principles of CTI delivered by the charity:

- Designed around a transition
- Time limited
- Community-based
- Developing social connection and capital
- Linking to long-term support (but not doing)
- Harm reduction / recovery orientated
- Limited focus
- Small caseloads
- Decreasing intensity / phased approach
- Frequent case reviews
- Team based weekly supervision

The intervention involves:

a) A pre-engagement phase:

Starting before discharge, an assessment panel meeting identifies those at higher risk of homelessness. The caseworker meets the client and begins a process of engagement and relationship building. They assess the client's needs and implement a tailored (and agreed) transition plan to link the client to

services and supports in the community. After the client is released from the institution, they are placed into tenancy (immediately or following a brief period in temporary accommodation).

b) 3 phases (lasting 3 months each):

Phase one ("Transition to the community"): an intense period of forming links and relationships and shared goal setting; aiming to improve crisis-resolution skills, provide support and advice tailored to their needs, and mediate any conflicts. This phase involves weekly home visits and other meetings with the client, with the client's caregivers, and community service providers.

Phase two ("Try out"): the caseworker monitors and adjusts the systems of support that were developed during phase one. This phase involves fewer meetings with the client, as the caseworker encourages the client to problem-solve and manage practical issues (e.g., sorting bills and general money/benefits management; living skills; social support and meaningful use of time; managing landlord relationship) with the help of community resources and family members, and intervenes only if the client is receiving inadequate support or if a crisis occurs.

Phase three ("Transfer of care"): the caseworker reduces the level of support and begins to 'step away'. They help the client to develop a plan to achieve long-term goals (e.g., employment, family reunification) and finalises the transfer of responsibilities to caregivers and community providers (i.e., the CTI intervention is terminated with support in place).

11.2 Compliance

Compliance to the intervention delivery will be measured as part of a fidelity assessment in the process evaluation using a previously developed CTI fidelity assessment tool (CTI-TS). A sample (n=24) of sessions will be observed/audio recorded for this purpose.

11.3 Training

Training will be provided for CTI staff members and recruiters on the recruitment and data collection processes. This will include trial specific training on the referral process, completion of screening logs, completion of the baseline measure and data entry on the database. A communication strategy will be developed to target Probation Officers and Community Offender Management teams to raise awareness of the trial and the referral process.

12 Trial procedures

The PHaCT trial will involve the following procedures:

1. Eligibility assessment

CTI staff will be informed of potential (i.e., those at risk of homelessness) recruits either by Probation Officers or the Community Offender Management team. On receiving details of potential individuals, they will screen these for eligibility at their weekly meetings and where individuals are eligible they will contact them and provide them with a brief verbal explanation of the trial with support of 1 summary information sheet. Where potential recruits express interest in taking part, the CTI staff member will contact the recruiting staff (i.e CRN/R&D/study staff) and arrange for them to attend the prison for a one off visit to take informed consent and complete the baseline assessment. Should the 45-minute prison visitor slot not allow sufficient time, the recruiter may split the baseline visit in two and book a subsequent visit. Should it not be possible to arrange a face-to-face meeting (i.e., in the case of a prison lockdown), alternative arrangements will be made by the CTI team for the recruiter to speak to the individual on the phone. This route will only be used as a back-up option.

2. Baseline visit

   a. Informed consent

At the baseline visit, the recruiter (if required) will provide the potential recruit with the full participant information sheet. Once a potential recruit has provided informed consent they will be regarded as a participant.

   b. Baseline CRF

After gaining informed consent, the recruiter will proceed with the baseline CRF. This will be completed on paper. The participant will be given the choice to either complete this on their own if they wish, or, with the support of the recruiter. See Figure 1 for full breakdown of baseline assessments (page 28).

   c. Allocation

Once the CRF has been completed the recruiter will proceed to reveal the allocation to the participant. Sealed envelopes containing the series of randomised allocations will be pre-prepared by trial statisticians for each prison and given to recruitment staff. Envelopes will be sequentially numbered, and the recruiter will be required to open the next envelope in the sequence. The contents of the envelope will state either ‘Usual care’ or ‘Critical Time Intervention’, along with the participants unique participant identification number (PID). The recruiter will then verbally explain (with use of a script), which group they have been allocated to and what the next step is for them (i.e., CTI team to make contact or follow usual prison procedure). The participant will have further opportunities to ask any

questions. The recruiter will then make a note of the participants PID number on the completed CRF and consent from and file these in separate concealed folders/envelopes.

3. <u>Data entry</u>

Within 48 hours of returning to the office, the recruiting staff will be required to enter the data on the bespoke online trial database and the paper CRF and consent form will be sent to CTR via registered mail for review and storage. Once the participants details and CRF responses have been entered the PHaCT trial team will receive an email notification of a new participant entry. This will be monitored by the Data Manager and where an individual has been randomised to the intervention arm, they will then inform the CTI team of the participants allocation. If recruitment is conducted by a trial team staff member, they will input the data after collection within 48 hours of collection.

4. <u>CTI Intervention</u>

Those participants who have been randomised to the intervention group will then be contacted by the CTI staff to proceed with the first stages of the intervention.

5. <u>Follow-up CRFs at 3-, 6-, 9- and 12-month</u>

All participants will be contacted to complete their follow-up assessments. Every effort will be made to complete all follow-ups in person with the same recruiter to aid in building rapport and maximising retention. However, where this is not possible, phone contact will be attempted by the research team. Where follow-up data collection is conducted in person the data will be collected on paper CRFs and where participants are contacted by phone (back-up option) data will be directly entered on the database. Follow-up visits will try to be arranged through the support of probation officers to maximise retention. At each follow-up assessment, the researcher will reaffirm their verbal consent to take part before completing the survey. Where it has not been possible to contact an individual after four attempts, they will be considered lost to follow-up at that timepoint. They will be contacted again for the next follow-up timepoint. Within 48 hours of returning to the office, the recruiter will be required to enter the data on the bespoke online trial database for each follow-up point and the paper CRF will be sent to CTR via tracked mail for review and storage.

6. <u>Interviews (RCT participants, staff/interventionists, non-RCT participants)</u>

    a. RCT Participant interviews

Of the participants who have consented to be interviewed, 12 intervention participants and 16 control participants will be sampled to take part in one-to-one semi-structured interviews with a member of the research team. Intervention participants will take part in repeated interviews split across 2

timepoints with the first interview conducted after phase 1 of the intervention and the 2nd interview after phase 2 of the intervention (total 24 interviews), however these will be augmented if required. Phase 1 interviews will focus on the recruitment methods and phase 2 interviews will focus on experiences of the intervention. Control participant interviews will take place after the final data collection timepoint at 12 months and will focus on participants experiences of taking part in the trial. Interviews will be arranged at a time and suitable place (i.e., at their probation officer meeting/phone call) for the participant to maximise retention and could be over the phone or in person. Before starting the interview, the researcher will explain what will happen and reaffirm their consent to take part. The interview will be audio recorded using an encrypted audio recorder.

b. Staff/interventionist interviews

Key prison/probation staff members and intervention staff (n=12) will be approached to take part in a one-to-one semi-structured interview with a member of the research team. Interviews will be arranged at a suitable time and place for the individual and where possible will be conducted face-to-face with phone interviews as a back-up option. Staff/interventionists will be provided with a separate information sheet and will be asked to provide written informed consent. The interview will be audio recorded using an encrypted audio recorder.

c. Non-RCT participant interviews

Individuals currently receiving CTI (n=12) will be approached to take part in an interview to assess the intervention. These will be one-to-one semi-structured interviews with a member of the research team. Interviews will be arranged at a suitable time and place for the individual and where possible will be conducted face-to-face in the CTI team offices with phone interviews as a back-up option. Non-RCT participants will be provided with a separate information sheet and will be asked to provide written informed consent. The interview will be audio recorded using an encrypted audio recorder.

7. <u>Session observations/audio recordings</u>

A sample of RCT participants (n=24) and non-RCT participants (n=12) of intervention sessions will be observed (maximum of 3 per person) where possible or audio recorded as a back-up to gain an insight into the delivery of the intervention. This will aid in the fidelity assessment of the intervention. Observations/audio recordings will be split across the different phases of the intervention. A fidelity assessment tool will be developed to support the observation data collection. Where sessions are audio recorded, research staff will use an encrypted recorder.

8. <u>Routine Data</u>

Where participants have consented to have their electronic routine data linked with health and/or criminal justice data, their details (i.e., name, address, gender, date of birth and NHS number where possible) will be sent to the relevant data providers (NHS England, SAIL and MoJ Criminal Justice dataLab) by the research team using a secure system (i.e., portal or secure email account) for linking.

12.1 Assessments

Figure 1. Schedule of enrolment, interventions and assessments[1]

**Procedures**	**Data collection timepoints**							
	**Screening**	**Baseline**	**Follow Up**				**Routine data**	**Rolling**
			**3 month**	**6 month**	**9 month**	**12 month**	**(SAIL/NHS England/MoJ DataLab)**	**CTI & Usual care costs**
Eligibility assessment	X	X						
Expression of interest	X							
Informed consent		X						
Affirmed consent			X	X	X	X		
Demographics		X						
Randomisation		X						
Delivery of intervention		X	X	X	X			
SF-12		x	x	x	x	X		
EQ-5D		x	x	x	x	x		
ICECAP-A		X	x	x	x	x		

[1] Taken from the HRA CTIMP protocol template (2016).

AUDIT-C		X	x	x	x	x		
Housing		X	x	x	x	x		
Resource use questionnaire		X	x	x	x	x	x	
Cost log (intervention and control)								X
Criminal Justice							x	
Participant interviews			Phase 1	Phase 2		Control		
Staff interviews			x	x	x	x		
Interventionist interviews			X	x	x	x		
Intervention observations & Fidelity assessment			x	x	x			
Safeguarding		X	X	X	X	X		
Withdrawal Checklist		X	X	X	X	X		

12.2 Follow-up

Participants will be followed up at 3- 6- 9- and 12-months post randomisation. This frequency of follow-up is used in RCTs with people experiencing homelessness to facilitate tracking of what can be a transitory population [44]. Follow-ups will be conducted in person wherever possible through the support of probation officer (i.e., at regular probation meetings). Four attempts will be made to follow

participants up at each timepoint and where it has not been possible to follow them up they will be considered to be loss to follow-up at that timepoint. The team will continue to follow-up participants at all remaining timepoints.

13 Safety reporting

We do not anticipate there to be any serious adverse events as a result of taking part of this trial or which can be attributed to the intervention. However, as this is a vulnerable population, they may experience some levels of distress due to their current situation which they may share with staff. For this reason, we will manage and record any safeguarding issues we become aware of during data collection and where possible during intervention delivery. To do this, we will include a safeguarding section in our participant information sheet and data collection forms where we will provide a number of contacts and links where individuals can seek additional support should they require this. Where there are any serious concerns, i.e., risk of harm or death to self or others, these will be reported immediately upon become aware of them to the Chief Investigator and escalated accordingly depending on the nature of the concerns (i.e., calling 999 where relevant or reporting these to the participants probation officers). Consent to escalate these will be collected. To keep a record of any safeguarding concerns, a 'safeguarding form' will be completed by the trial team/CRN/R&D staff within 24 hours of becoming aware of the concerns. Reported safeguarding concerns will be reviewed at monthly TMG meetings and signed off by the Chief investigator. Regular debriefing sessions will be held with the recruiters who are involved with data collection to ensure they are well supported. Where any face-to-face data collection will be conducted (i.e., for baseline recruitment, follow-up data collection, interviews or observations) these will take place in suitable public venues and no home data collection will take place. Data collection in the prison will be completed by named recruiter and will follow the visitor system whereby they will be escorted and accompanied by prison staff key holders/CTI staff.

Safeguarding concerns may include:

- Participants/others safety
- Participants declined mental health
- Research staff safety
- Research staff wellbeing

For any lone working during the qualitative data collection we will follow the guidelines set out in the SOP on lone working (SOP/003/12) to ensure safety of researchers.

13.1 Definitions

Term	Definition
Safeguarding concern	Any issue that includes but is not necessarily limited to:<br>• Self-reported immediate self-harm or suicide plans<br>• Self-reported immediate plans to harm others

13.2 Reporting process

1. Within 24 hours of being made aware of safeguarding concern this should be reported to the trial team.
2. Where possible (i.e., access to the trial database) the individuals should directly complete a safeguarding form, however if reported by the CTI team, this should be reported to the trail team by phoning or emailing the trial administrator. No identifiable information should be used and participants should only be referred to by their PID number.
3. The trial team will review/enter details on the safeguarding form. Where external input is needed, i.e., probation officer etc. this will be sought.
4. Safeguarding forms will be reviewed by the Chief Investigator and signed off once appropriately resolved.
5. Safeguarding data will be collated onto a log by the Data Manager and reviewed at monthly TMG meetings.

14 Statistical considerations

14.1 Randomisation

Randomisation will be completed by a trial statistician on a 1:1 ratio and stratified by prison, using random blocks. A randomisation protocol will be developed separately and will include testing of the process and the system. Due to the restrictions of using electronic equipment during recruitment, randomisation will be completed using concealed paper envelopes. Each site will be provided with a box containing 60 sealed sequentially numbered envelopes (total 4 boxes). Envelopes will contain the arm allocation and PID number. Boxes and all envelopes will be clearly labelled with the prison name and sequence number. Training will be provided to site staff to outline the randomisation method. Prepared randomisation boxes will be checked by a member of the trial team to ensure the correct sequence is followed before being sent to sites. Randomisation method will be clearly documented in the randomisation protocol. Randomisation will be checked by TM or DM to ensure correct allocation.

14.2 Blinding

Allocations will be revealed after the baseline assessment, therefore at baseline participants and staff will be blind to group allocation. However, once randomised it will not be possible for participants or staff members to be blind as they will either receive the intervention or receive usual care. However, the trial statistician and health economist will remain blinded to group allocation during analysis. It will not be possible to blind the qualitative researcher.

14.3 Sample size

No power calculations have been performed as our aim is to evaluate acceptability of the intervention, not to estimate effectiveness. This study will determine rates of recruitment, response and retention, and the distribution of the indicative primary outcome measures in a full-scale RCT, to inform a fullscale trial sample size calculation. Given there is capacity to deliver the intervention to 120 participants per year, we aim to recruit 80 participants (40 per arm) which allows us to estimate a conservative recruitment rate of 50% within a 95% confidence interval (CI) of ±11% (75% rate ±9.5%).

14.4 Missing, unused & spurious data

The rate of completeness for baseline and follow-up measures will be described. No imputation will be performed.

14.5 Procedures for reporting deviation(s) from the original SAP

Any deviations from the original statistical analysis plan (SAP) will be submitted as substantial amendments where applicable and recorded in subsequent versions of the protocol and SAP.

14.6 Termination of the trial

There will be no formal ‘stopping rules’ or ‘discontinuation criteria’ for individual participants, parts of study and entire study.

14.7 Inclusion in analysis

The trial population will be all participants who are eligible and consent to participate in the trial.

15 Analysis

15.1 Main analysis

The reporting of findings will be in accordance with the CONSORT guidelines for pilot RCTs [47]. The primary analysis of the pilot RCT will determine whether the prespecified progression criteria to a fullscale trial are met. All outcomes related to feasibility will be reported as point estimates with 95% confidence intervals (CIs).

Recruitment, baseline response, randomisation, follow-up at each time point, and consent to routine data linkage will be summarised in a CONSORT flow diagram. We will tabulate demographic characteristics of participants by study arm using descriptive statistics: means and standard deviations (SDs)(or medians and interquartile (IQR) ranges, as appropriate) for continuous outcomes, frequencies and percentages for categorical outcomes.

We will examine rates of completion of outcomes at each follow-up time point. We will pilot the analyses of effectiveness outcomes that would be done in a full-scale trial. Each outcome will be summarised at each time point (number (%), mean (SD), median (IQR)) and will be analysed in a separate multi-level regression model following the intention to treat approach and using data at baseline and follow up. The model will include fixed effects for intervention group, assessment time point (3, 6, 9, 12 months) and an interaction term between group and time. The model will allow us to examine the change in intervention over time compared to changes in the control arm. These estimates (odds ratios, mean differences) will be adjusted for participants nested within prison and will be presented alongside 95% CIs.

As this is a pilot trial, not powered for effectiveness, no hypothesis testing will be performed and no p-values presented. We will also estimate the clustering of outcomes within prisons and partial clustering of prisoners those imprisoning caseworkers (CTI arm only) by estimating intra-cluster correlation coefficients (with 95% CIs). Full details of the statistical analysis will be detailed in the statistical analysis plan (SAP). All analyses will be performed in Stata or R.

15.1.1 Sub-group & interim analysis No
sub-group analyses are planned.

15.2 Qualitative analysis

All interviews will be transcribed verbatim (audio recordings will be uploaded and transcribed by an approved Cardiff University supplier via a secure portal for the purpose of transcription and deleted from the portal once transcription is complete) and analysed using Thematic Analysis [48]. A coding framework will be developed which will be closely aligned with the trial specific process evaluation framework and data will be categorised accordingly. Commonly expressed themes as well as unusual cases will be identified. Intervention delivery observations/audio recordings will be coded against key intervention components to assess fidelity of the intervention delivery. All interview transcripts will be checked for accuracy/anonymisation and 20% will be double-coded to check for reliability of the coding framework.

The qualitative analysis will then feed into the overall process evaluation which will be conducted in accordance with the MRC guidance [45] and will use a mixed methods approach. The aim of the process evaluation will be to report on each of the process evaluation components (Reach, Recruitment and Retention, Fidelity and Acceptability, Compliance, Contamination and Context) [45]. Fidelity will be assessed with support from a previously developed CTI fidelity assessment tool (CTITS) [46]. Data sources will be mapped against each of these components and data will be triangulated (where relevant) to provide a detailed overview of participants and staff experiences and acceptability of the intervention as well as the delivery methods and fidelity of delivery.

This analysis will ultimately inform understanding of acceptability, views and experiences of the intervention and acceptability of trial, to inform acceptability of a full-scale trial.

15.3 Economic evaluation

The broader health and social impacts of the intervention, and associated costs will be considered to inform an economic evaluation in a full-scale trial. In particular, we will: test the feasibility of data collection instruments in capturing costs associated with participants' short-term use of health and community services and broader costs (e.g., criminal justice, welfare costs); test feasibility of using cost logs to capture the costs to deliver the intervention (e.g., staff costs); examine the distribution of the health-related quality of life and capability measures (EQ-5D and ICECAP-A has previously been successfully used in the people experiencing homelessness population [5, 6, 49]) and measure its association with housing stability, physical and mental health. Descriptive statistics (including levels of missing data) for each outcome and cost variable will be reported at each data collection time point and for each intervention group. The patterning and percentage of missing data will inform likely analytical strategy in full-scale trial (e.g., whether multiple imputation is necessary). The distribution of outcome and cost variables will inform likely distributional assumptions to be made when propagating uncertainty through the cost-effectiveness analyses.

An exploratory cost-consequences analysis [50] will report costs alongside consequences for intervention and control groups (this will involve participation of PPI representatives to ensure all relevant costs and consequences are captured). Although cost-effectiveness analyses will be considerably uncertain at this pilot trial stage, we will conduct a short-term (1 year time horizon) costutility analysis. Kaplan-Meier estimates of survival probabilities will be combined with the repeated measurements of costs and EQ-5D to obtain mean costs and quality adjusted life years (QALYs) for both intervention and control groups (allowing estimation of the incremental cost-effectiveness ratio) [51]. Scenario sensitivity analysis [52] will be used to identify prices of the CTI intervention at which it would

be cost-effective (using NICE threshold) given a range of plausible short-term QALY gains. We will use the cost-utility analysis results to inform value of information (VOI) [53] analyses. These VOI analyses will also assist in identifying the likely key parameters that will drive decision uncertainty and therefore help in planning the design of the full-scale trial.

A review of existing evidence will assist in: 1) identifying suitable modelling frameworks to assess the cost-effectiveness of the CTI intervention over the long-term; 2) identifying the key sources of uncertainty in relation to the probability of the intervention being cost-effective when a long-term time horizon is used. This review and the pilot economic evaluation outlined above will be used in combination to develop an economic conceptual model [54] for the long-term, to be developed at full-scale trial stage. The process evaluation, cost consequences analysis and cost-utility analysis in this pilot trial will inform the optimal time horizon for the economic evaluation alongside the full-scale trial. We will explore using the linked data sources (see section 15.4) to inform longer-term costeffectiveness modelling in the full-scale trial stage.

15.4 Routine data analysis

We will review existing routine data sources that could be used to assess outcomes at the main stage trial. Provisional outcomes of interest (see logic model in Appendices) include housing stability, criminal justice contacts, alcohol and substance use, and mental health. We will attempt to harmonize potential data sources so that similar outcomes can be measures from across the devolved UK nations. based on this review, data owners will be contacted to assess whether data are accessible for linkagebased studies, and any practical and financial implications if accessing these data. We will also work with data owners to develop trial materials, such as consent questions and participant information sheets, which meet their requirements to enable access to data for the main stage trial without the need for these to be further developed. The review will also seek to explore potential codes within data sources that align with measurable outcomes of interest, i.e., diagnosis codes for hospitalisations due to substance use.

In tandem with this data exploration and engagement activity, we will also be conducting test data linkages to healthcare data sets in England and Wales, and criminal justice data. Test linkages will allow us to assess the percentage of participants who consent to have their data linked and the percentage of data records which can be linked successfully with these data providers. We will generate summary statistics of the completeness of data needed to undertake linkage, i.e., percentage of people providing personal details. As each data owner uses different data matching methods when extracting records, information on the proportion of people from the original trial cohort who we are able to link to will

aid in assessing the extent to which linkage at a full trial will generate samples with enough power to detect intervention effects.

De-identified healthcare data will be accessed via NHS England and the Secure Anonymised Information Linkage (SAIL) databank, in England and Wales respectively. Applications to access healthcare data will be made to NHS England via the Data Access Request Service and to SAIL via their Information Governance Review Panel. Information requested from NHS England and SAIL will be limited for the purposes of a test linkage to records related to periods spent in hospital, use of accident and emergency services, outpatients' services, and death records [55]. We will request data from 5years prior to enrolment in the study, to assess the potential of linkage to develop baseline healthcare use estimates, up to the latest data available, to assess the use of linkage to evaluate difference in health outcomes between study arms.

We will also seek to link to criminal justice data via the Ministry of Justice Data Lab ('the Lab'). The Lab is a service operated by the Ministry of Justice that links details of clients engaging with third sector organisations and provides summary reports of re-offending rates for clients–compared to a matched comparison group that the Lab generates form the wider prisoner population. As the Lab's services may conflict with trial procedures, such as having two trial study arms and the requirement that summary reports be published on the MoJ website, the test linkage to criminal justice data may not occur if deemed incompatible with study goals.

To link records, personal details will need to be shared with data owners, including name, date of birth, gender, and postcode. It may help data owners if unique identification numbers used by their computer systems are provided; therefore, Police National Computer number and NHS number will also be collected and shared with the appropriate data owner. We will use consent questions and participant information materials based on previous studies from the CTR.

The trial Data Manager will be responsible for the processing of personal data prior to sending it to relevant data owners. Only pre-approved secure methods will be used to transfer personal data between the CTR and data owners. Trial researchers will have access to either aggregated data reports (in the case of the MoJ Data Lab) or de-identified data which will be accessed via a secure gateway (SAIL Gateway)—see data flow diagrams in Appendices.

16 Data Management

A Data Management Plan (DMP) and Qualitative data collection and Analysis Plan (QAP) will be developed to outline the different types of data being collected, collection methods, processing of

data, data storage and data sharing. The DMP will include details of the management of the following source data:

Trial data	*Source data*								
	Screening	*CRF*	*Electronic routine health data*	*Aggregated criminal justice data*	*Participant interviews*	*Staff interviews*	*Observational notes/audio recordings*	*Safeguarding*	*Withdrawal*
Recruitment	*x*				*X*	*X*			
Health		*X*	*X*		*X*	*X*			
Mental health		*X*	*X*		*X*	*X*			
Substance misuse		*x*	*x*		*X*	*X*			
Police & MoJ contact				*x*	*X*	*X*			
Intervention Engagement					*X*	*x*	*x*		
Intervention content delivery					*X*	*X*	*X*		
Safeguarding								*X*	
Withdrawal									*x*

Source Data is defined as *"All information in original records and certified copies of original records of clinical findings, observations or other activities in a trial necessary for the reconstruction and evaluation of the trial. Source data are contained in source documents."* There is only one set of source data at any time for any data element, as defined in site source data agreement.

16.1 Data collection

Data will be collection directly from participants, staff and through secure data providers. Data will be collected either via paper, electronic, audio recordings or observation notes form the intervention delivery. Any paper based data collection will be securely transported and entered on the trial database on return to the office, personal details will be shared securely with data providers, audio recordings will be uploaded to a secure portal for secure transcription and any hand written observational notes will be word processed as soon as possible.

stor16.2 Completion of CRFs

16.2.1 Paper CRFs

Data will be collected via specifically designed trial paper CRFs. Paper CRFs will be designed with support from the trial PPI lead to sure it is easy to follow. The recruiter (CRN/R&D staff/research team) will securely transport CRFs. Once the recruiters return to the office they will enter the data directly onto the trial database. This will reduce any unnecessary data queries. Once the data has been entered, paper CRFs will be stored in the site file and later returned to the trial team in registered post. CRF pages and data received by the trial team from participating trial sites will then be checked against the database for missing, illegible or unusual values (range checks) and consistency over time. If missing or questionable data are identified, a data query will be raised on a data clarification form. The data clarification form will be sent to the relevant participating site. The site shall be requested to respond to the data query on the data clarification form. The case report form pages should not be altered. All answered data queries and corrections should be signed off and dated by a delegated member of staff at the relevant participating site. Where common queries or errors arise on data entry these will be entered onto a self-evident correction log with agreement from TMG members. The completed data clarification form should be returned to the trial team and a copy retained at the site along with the participants' CRFs. The trial team will send reminders for any overdue data. It is the site's responsibility to submit complete and accurate data in timely manner.

16.2.2 Electronic CRFs

An electronic bespoke database will be developed using Qualtrics to capture data collected via CRFs, withdrawal and any safeguarding concerns. This is a secure encrypted system and will be hosted on the secure Cardiff University server accessed through a password protected website and only named individuals (outlined on a trial and site delegation log) will have access. All individuals who have access will be trained in Good Clinical Practice (GCP) and data handling. A training log will keep record of this.

Electronic data entry will be quality controlled through the use of appropriate skips and parameters and the system will be tested prior to the trial opening for recruitment.

17 Protocol/GCP non-compliance

Any reports of any non-compliance to the trial protocol or the conditions and principles of Good Clinical Practice need to be reported to the Chief Investigator and Trial Manager in writing as soon as they become aware of it. All serious non-compliances to be reported to the Sponsor representative.

18 End of Trial definition

The end of the trial is defined as the date of final data capture to meet the trial endpoints. In this case end of trial is defined as the 12 months-post randomisation follow-up timepoint.

The trial team will notify the REC of the end of the trial within 90 days of its completion or within 15 days if the trial is terminated early.

19 Archiving

The Trial Master File (TMF) containing essential documents will be archived at an approved external storage facility for a minimum of 15 years. The CTR will archive the TMF on behalf of the Sponsor according to the CTR Archiving Standard Operating Procedure. Any data shared with and held at University of Glasgow for the purposes of the health economic analysis will be stored securely according with University of Glasgow policy for a minimum of 10 years.

20 Regulatory Considerations

20.1 Ethical and governance approval

This protocol has approval by the sponsor (University of Glasgow) and Wales Research Ethics Committee (REC) 3 that is legally "recognised" by the United Kingdom Ethics Committee Authority for review and approval.

This trial protocol will be submitted through the relevant permission system for global governance review.

Approval will be obtained from each prison, CRN and R&D board who will consider local governance requirements and site feasibility. The Research Governance approval of the host organisation will be obtained before recruitment of participants commences.

20.2 Data Protection

The trial team will act to preserve participant confidentiality and will not disclose or reproduce any information by which participants could be identified, except where specific consent is obtained. Data will be stored in a secure manner and will be registered in accordance with the UK General Data Protection Regulation. The independent data controllers for this trial are the University of Glasgow and Cardiff University.

20.2.1 Data sharing

Data will be shared with the University of Glasgow for the purpose of conducting the Health Economic analysis. De-identified data may be made available following review against the CTR policies on data sharing and with an agreed/signed data sharing agreement.

20.3 Indemnity

The Sponsor has appropriate insurance to indemnify the site against claims arising from the acts and/or omissions of the Sponsor or its employees in connection with the Trial (including the design of the Protocol to the extent that the Protocol was designed solely by the Sponsor and co-investigators and that the Site has adhered to the approved version of the Protocol) save to the extent that any such claim is the result of negligence on the part of the Site or its employees.

20.4 Trial sponsorship

University of Glasgow will act as Sponsor for the trial. Delegated responsibilities will be assigned to the Centre for Trials Research (CTR), Cardiff University in order to manage and run the trial and detailed responsibilities will be outlined in the Roles and Responsibilities Log.

20.5 Funding

The Trial has been funded by the National Institute of Healthcare Research (NIHR) Ref: NIHR134281 with a start date of 1st June 2022.

21 Trial management

21.1 TMG (Trial Management Group)

The TMG will meet on a monthly basis online with video conferencing details provided. Face to face meetings will be arranged throughout the duration of the trial, with the option of these being hybrid for those wishing to attend virtually instead. Meeting documentation will be sent via email one week prior to each meeting to allow members time to read any required materials. TMG members will be

required to input into the study developments throughout all stages. TMG members will be required to sign up to the remit and conditions as set out in the TMG Charter.

21.2 TSC (Trial Steering Committee)

The TSC will meet on 3 occasions or more often if deemed necessary. The TSC will consist of external independent experts within prison or homelessness fields and will also include a trial statistician. The TSC will have oversight of the trial development and will support the development of the trial in any which way deemed appropriate. They will additionally take on the responsibility of the Data Monitoring Committee (DMC).

TSC members will be required to sign up to the remit and conditions as set out in the TSC and DMC Charters.

22 Quality Control and Assurance

22.1 Monitoring

The trial risk assessment has been used to determine the intensity and focus of central and on-site monitoring activity in the PHaCT trial. Low monitoring levels will be employed and are fully documented in the trial monitoring plan. This will include monitoring of the trial screening/recruitment process, randomisation and PID allocation as well as monitoring of data entry. Investigators should agree to allow trial related monitoring, including audits and regulatory inspections, by providing direct access to source data/documents as required. Participant consent for this will be obtained.

Findings generated from on-site and central monitoring will be shared with the Sponsor, Chief Investigator, & CRN/R&D.

22.2 Audits & inspections

The trial may be audited and inspected by the University of Glasgow under their remit as Sponsor. The study may be audited by NHS England Audit Team.

23 Publication policy

A publication policy will be developed in line with CTR processes. This will outline planned publications and presentations, targeted journals and conferences and writing groups. All publications and presentations relating to the trial will be authorised by the Trial Management Group. All appropriate acknowledgment statements will be used for any publications.

24 Milestones

A study Gantt will be used to keep track of key study timelines. This will be reviewed at regular meetings and kept up to date by the trial manager.

25 References

1. B. Volker, R. De Cuyper, G. Mollenhorst, A. Dirkzwager, P. van der Laan, and P. Nieuwbeerta, "Changes in the social networks of prisoners: A comparison of their networks before and after imprisonment," Social Networks, vol. 47, pp. 47-58, 2016.
2. S. Fazel, J. R. Geddes, and M. Kushel, "The health of homeless people in highincome countries: descriptive epidemiology, health consequences, and clinical and policy recommendations," The Lancet, vol. 384, no. 9953, pp. 1529-1540, 2014.
3. P. Goering, G. Tolomiczenko, T. Sheldon, K. Boydell, and D. Wasylenki, "Characteristics of persons who are homeless for the first time," Psychiatric Services, vol. 53, no. 11, pp. 14721474, 2002.
4. M. B. Kushel, S. Perry, D. Bangsberg, R. Clark, and A. R. Moss, "Emergency department use among the homeless and marginally housed: results from a community based study," American journal of public health, vol. 92, no. 5, pp. 778-784, 2002.
5. D. Lewer et al., "Health-related quality of life and prevalence of six chronic diseases in homeless and housed people: a cross-sectional study in London and Birmingham, England," BMJ Open, vol. 9, no. 4, p. e025192, 2019.
6. S. Sun, R. Irestig, B. Burström, U. Beijer, and K. Burström, "Health-related quality of life (EQ5D) among homeless persons compared to a general population sample in Stockholm County, 2006," Scandinavian journal of public health, vol. 40, no. 2, pp. 115-125, 2012.
7. R. W. Aldridge et al., "Morbidity and mortality in homeless individuals, prisoners, sex workers, and individuals with substance use disorders in high-income countries: a systematic review and meta-analysis," The Lancet, vol. 391, no. 10117, pp. 241-250, 2018.
8. S. W. Hwang, E. Gogosis, C. Chambers, J. R. Dunn, J. S. Hoch, and T. Aubry, "Health status, quality of life, residential stability, substance use, and health care utilization among adults

applying to a supportive housing program," Journal of Urban Health, vol. 88, no. 6, pp. 10761090, 2011.

9. J. Travis, A. L. Solomon, and M. Waul, "From prison to home: The dimensions and consequences of prisoner reentry," 2001.
10. D. A. Snow, S. G. Baker, and L. Anderson, "Criminality and homeless men: An empirical assessment," Social Problems, vol. 36, no. 5, pp. 532-549, 1989.
11. K. Reeve, "The hidden truth about homelessness," 2011.
12. D. Dixon, "Economic benefits of supporting homeless young people," Sykes H Youth Homelessness,. Courage and Hope, Melbourne University Press, Melbourne, 1993.
13. N. Pleace, "At what cost? An estimation of the financial costs of single homelessness in the UK," Crisis, July, 2015.
14. P. T. Mackie, et al., "Nations apart? Experiences of single homeless people across Great Britain. London: Crisis." ed, 2014. https://www.crisis.org.uk/endinghomelessness/homelessness-knowledge-hub/types-ofhomelessness/nations-apartexperiences-of-single-homeless-people-across-great-britain2014/
15. "Offender Rehabilitation Act 2014." The National Archives. https://www.legislation.gov.uk/ukpga/2014/11/contents/enacted
16. National Pathway for Homelessness Services to Children, Young People and Adults in the Secure Estate. 2015 [Online] https://gov.wales/sites/default/files/publications/2019-03/homelessness-services-for-children-young-people-and-adults-in-the-secure-estate_0.pdf
17. "Homelessness Reduction Act 2017." The National Archives. https://www.legislation.gov.uk/ukpga/2017/13/contents/enacted
18. Her Majesty's Inspectorate of Probation, "Accommodation and support for adult offenders in the community and on release from prison in England," 2020.
19. D. Ponka et al., "The effectiveness of case management interventions for the homeless, vulnerably housed and persons with lived experience: A systematic review," (in eng), no. 19326203 (Electronic), 2020.
20. J. Draine and D. B. Herman, "Critical time intervention for reentry from prison for persons with mental illness," (in eng), no. 1075-2730 (Print), 2007.

21. D. B. Herman, S. Conover, P. Gorroochurn, K. Hinterland, L. Hoepner, and E. S. Susser, "Randomized trial of critical time intervention to prevent homelessness after hospital discharge," Psychiatric services, vol. 62, no. 7, pp. 713-719, 2011.
22. E. Susser, E. Valencia, S. Conover, A. Felix, W.-Y. Tsai, and R. J. Wyatt, "Preventing recurrent homelessness among mentally ill men: a" critical time" intervention after discharge from a shelter," American Journal of Public Health, vol. 87, no. 2, pp. 256-262, 1997.
23. W. J. Kasprow and R. A. Rosenheck, "Outcomes of critical time intervention case management of homeless veterans after psychiatric hospitalization," Psychiatric Services, vol. 58, no. 7, pp. 929-935, 2007.
24. M. Shinn, J. Samuels, S. N. Fischer, A. Thompkins, and P. J. Fowler, "Longitudinal Impact of a Family Critical Time Intervention on Children in High-Risk Families Experiencing Homelessness: A Randomized Trial," (in eng), no. 1573-2770 (Electronic), 2015.
25. R. Bai, C. Collins, R. Fischer, and D. Crampton, "Family critical time intervention with housing unstable, child welfare-involved families: Service providers' and families' experiences with the phases," Children and Youth Services Review, vol. 119, p. 105592, 2020/12/01/ 2020, doi: https://doi.org/10.1016/j.childyouth.2020.105592.
26. J. Shaw et al., "Critical time Intervention for Severely mentally ill Prisoners (CrISP): a randomised controlled trial," 2017.
27. C. Lennox et al., "Critical Time Intervention: a qualitative study of the perspectives of prisoners and staff," The Journal of Forensic Psychiatry & Psychology, vol. 31, no. 1, pp. 76-89, 2020/01/02 2020, doi: 10.1080/14789949.2019.1665699.
28. D.-Y. Kim and S. M. Gayadeen, "Use of Critical Time Intervention (CTI) to Promote Continuity of Reintegration After Incarceration," Criminal Justice Review, vol. 46, no. 3, pp. 281-303, 2021/09/01 2020, doi: 10.1177/0734016820958139.
29. A. J. Baxter, E. J. Tweed, S. V. Katikireddi, and H. Thomson, "Effects of Housing First approaches on health and well-being of adults who are homeless or at risk of homelessness: systematic review and meta-analysis of randomised controlled trials," J Epidemiol Community Health, vol. 73, no. 5, pp. 379-387, 2019.
30. R. de Vet, S. N. van Luijtelaar Mj Fau - Brilleslijper-Kater, W. Brilleslijper-Kater Sn Fau - Vanderplasschen, M. D. Vanderplasschen W Fau - Beijersbergen, J. R. L. M. Beijersbergen Md Fau - Wolf, and J. R. Wolf, "Effectiveness of case management for homeless persons: a systematic review," (in eng), no. 1541-0048 (Electronic), 2013.

31. D. Herman, A. Opler L Fau - Felix, E. Felix A Fau - Valencia, R. J. Valencia E Fau-Wyatt, E. Wyatt Rj Fau - Susser, and E. Susser, "A critical time intervention with mentally ill homeless men: impact on psychiatric symptoms," (in eng), no. 0022-3018 (Print), 2000.
32. R. de Vet et al., "Critical time intervention for homeless people making the transition to community living: a randomized controlled trial," American journal of community psychology, vol. 60, no. 1-2, pp. 175-186, 2017.
33. D. A. M. Lako et al., "The effectiveness of critical time intervention for abused women leaving women's shelters: a randomized controlled trial," (in eng), Int J Public Health, vol. 63, no. 4, pp. 513-523, 2018, doi: 10.1007/s00038-017-1067-1.
34. J. Samuels, P. J. Fowler, A. Ault-Brutus, D.-I. Tang, and K. Marcal, "Time-limited case management for homeless mothers with mental health problems: Effects on maternal mental health," Journal of the Society for Social Work and Research, vol. 6, no. 4, pp. 515-539, 2015.
35. A. Tomita and D. B. Herman, "The impact of critical time intervention in reducing psychiatric rehospitalization after hospital discharge," (in eng), Psychiatr Serv, vol. 63, no. 9, pp. 935-937, 2012, doi: 10.1176/appi.ps.201100468.
36. L. Dixon et al., "Use of a Critical Time Intervention to Promote Continuity of Care After Psychiatric Inpatient Hospitalization," Psychiatric Services, vol. 60, no. 4, pp. 451-458, 2009/04/01 2009, doi: 10.1176/ps.2009.60.4.451.
37. C. Clark, C. C. Guenther, and J. N. Mitchell, "Case management models in permanent supported housing programs for people with complex behavioral issues who are homeless," Journal of Dual Diagnosis, vol. 12, no. 2, pp. 185-192, 2016.
38. K. Jones et al., "Cost-effectiveness of critical time intervention to reduce homelessness among persons with mental illness," Psychiatric Services, vol. 54, no. 6, pp. 884-890, 2003.
39. S. Sheikh and D. Teeman, "A rapid evidence assessment of what works in homelessness services," ed: UK. Retrieved from www. pc. gov. au/rogs, 2018.
40. E. A.-O. X. Tweed et al., "Health of people experiencing co-occurring homelessness, imprisonment, substance use, sex work and/or severe mental illness in high-income countries: a systematic review and meta-analysis. LID - jech-2020-215975 [pii] LID -10.1136/jech-2020215975 [doi]," (in eng), no. 1470-2738 (Electronic), 2021.
41. D. Herman, A. Conover S Fau - Felix, A. Felix A Fau - Nakagawa, D. Nakagawa A Fau - Mills, and D. Mills, "Critical Time Intervention: an empirically supported model for preventing homelessness in high risk groups," (in eng), no. 0278-095X (Print), 2007.

42. C. L. Caton, A. Wyatt Rj Fau - Felix, J. Felix A Fau - Grunberg, B. Grunberg J Fau - Dominguez, and B. Dominguez, "Follow-up of chronically homeless mentally ill men," (in eng), no. 0002953X (Print), 1993.
43. D. Herman and J. Mandiberg, "Critical Time Intervention: Model Description and Implications for the Significance of Timing in Social Work Interventions," Research on Social Work Practice - RES SOCIAL WORK PRAC, vol. 20, 09/01 2010, doi: 10.1177/1049731509360667.
44. T. Aubry et al., "A randomized controlled trial of the effectiveness of Housing First in a small Canadian City," BMC Public Health, vol. 19, no. 1, p. 1154, 2019/08/22 2019, doi: 10.1186/s12889-019-7492-8.
45. G. F. Moore et al., "Process evaluation of complex interventions: Medical Research Council guidance," BMJ, vol. 350, 2015.
46. T. F. C. da Silva, G. M. Lovisi, and S. Conover, "Developing an instrument for assessing fidelity to the intervention in the Critical Time Intervention-Task Shifting (CTITS)—Preliminary report," Archives of Psychiatry and Psychotherapy, vol. 16, no. 1, pp. 55-62, 2014, doi: 10.12740/APP/23279.
47. S. M. Eldridge et al., "CONSORT 2010 statement: extension to randomised pilot and feasibility trials," (in eng), no. 1756-1833 (Electronic), 2016.
48. V. Braun and V. Clarke, "Using thematic analysis in psychology," Qualitative research in psychology, vol. 3, no. 2, pp. 77-101, 2006.
49. N. Kozloff, A. D. Pinto, V. Stergiopoulos, S. W. Hwang, P. O'Campo, and A. M. Bayoumi, "Convergent validity of the EQ-5D-3L in a randomized-controlled trial of the Housing First model," BMC health services research, vol. 19, no. 1, p. 482, 2019.
50. NICE, "Methods for the Development of NICE Public Health Guidance", ed. London: National Institute for Clinical Excellence, 2012.
51. R. Etzioni, M. Urban N Fau - Baker, and M. Baker, "Estimating the costs attributable to a disease with application to ovarian cancer," (in eng), no. 0895-4356 (Print), 1996.
52. N. Latimer et al., "HEDS Discussion Paper 11/10 - Modelling the cost effectiveness of a potential new neck collar for patients with motor neurone disease," University of Sheffield, 2011.
53. E. Fenwick et al., "Value of Information Analysis for Research Decisions—An Introduction: Report 1 of the ISPOR Value of Information Analysis Emerging Good Practices Task Force," Value in Health, vol. 23, no. 2, pp. 139-150, 2020/02/01/ 2020, doi: https://doi.org/10.1016/j.jval.2020.01.001.

54. H. Squires, J. Chilcott, R. Akehurst, J. Burr, and M. P. Kelly, "A framework for developing the structure of public health economic models," Value in Health, vol. 19, no. 5, pp. 588-601, 2016.
55. M. Farrell and J. Marsden, "Acute risk of drug-related death among newly released prisoners in England and Wales," Addiction, https://doi.org/10.1111/j.1360-0443.2007.02081.x vol. 103, no. 2, pp. 251-255, 2008/02/01 2008, doi: https://doi.org/10.1111/j.13600443.2007.02081.x.

26 Appendices

26.1 Logic model

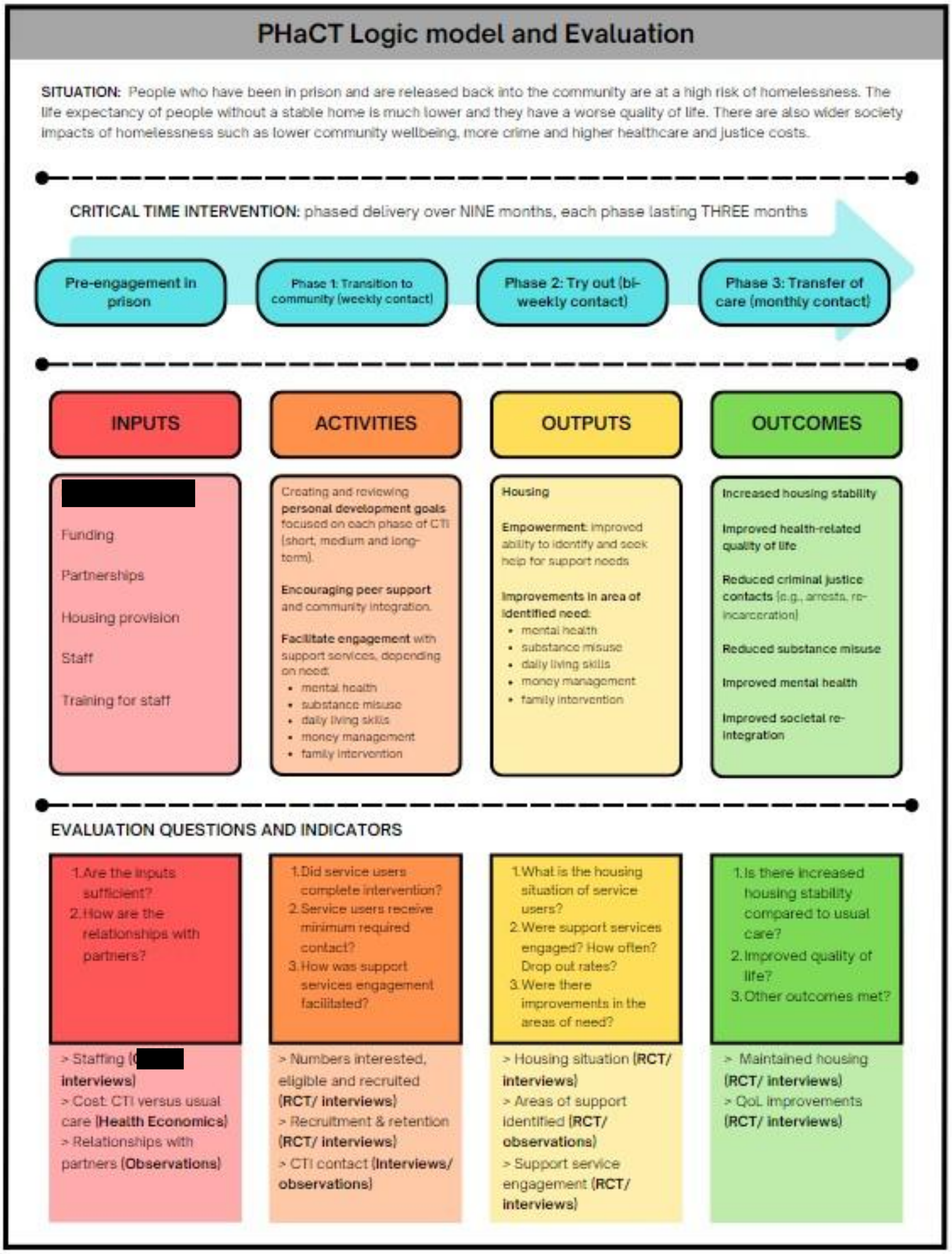

Supplement: online supplemental file 1 [file bmjopen-15-12-s001.pdf]
