# Supplementary material for: A pilot randomised controlled trial of a critical time intervention for people leaving prison: findings from an integrated process evaluation
Source: BMJ Open. 2025 Dec 17;15(12):e097761. doi: 10.1136/bmjopen-2024-097761 (PMC12716555; doi:10.1136/bmjopen-2024-097761)
Supplement: Supplementary file 2 [file bmjopen-2024-097761.reviewer_comments.pdf]

PEER REVIEW HISTORY

BMJ Open publishes all reviews undertaken for accepted manuscripts. Reviewers are asked to complete a checklist review form and are provided with free text boxes to elaborate on their assessment. These free text comments are reproduced below.

ARTICLE DETAILS

Title (Provisional)

A pilot randomised controlled trial of a Critical Time Intervention for people leaving prison: findings from an integrated process evaluation

Authors

Williams, Adam Dale Newman; Jacob, Nina; Moriarty, Yvonne; Madoc-jones, Iolo; Fitzpatrick, Suzanne; Mackie, Peter; Thomas, Ian; Grozeva, Detelina; Lloyd, Barry; Deidda, Manuela; Achiaw, Samuel Owusu; Lewis, Kelly; Cannings-John, Rebecca; Katikireddi, Srinivasa Vittal; White, James; Lewsey, Jim

---

VERSION 1 - REVIEW

**Reviewer**	**1**
**Name**	**Sondhi, Arun**
**Affiliation**	**Therapeutic Solutions**
**Date**	**10-Feb-2025**
**COI**	**None**

The paper addresses a potentially crucial intervention - CTIs - focusing on a core component underpinning a person's rehabilitation upon leaving prison: access to safe and secure housing. This is widely written about in the academic and policy-based literature.

The paper is well-written and structured, although I have doubts about integrating the process parts and details about developing the trial to ensure a clear narrative and focus. The quotes are also well-chosen and often illuminating. However, I have a few suggestions for the authors to consider.

I was interested in the choice of model. What is the genesis of this model in this context? Variations of CTI exist, and in the US, it feels like this model is aligned to 'Rapid-Rehousing' approaches, which would be worth including as an overview. Key to Rapid Housing is the leveraging of financial support (e.g. in the US, the Recovery and Reinvestment Act) that allocates resources to support the development of this model. I'd recommend reviewing Byrne’s (2022) overview of this and systematic review of the findings. An awareness of this literature would help contextualize the findings – especially regarding resourcing appropriate housing for people leaving prison (and its cultural applicability in a UK context).

The introduction must be clear that the location is England and Wales for an international audience, and some references to the broader policy context should be included. For example, in England and Wales, prior Transforming Rehabilitation and Through-the-Gate initiatives would fall within the scope of the CTI model (and the Drug Intervention Programme before that). The continuity of policy is worth discussing in the introduction and later. CTI could be argued to be a version of previous initiatives. A wider discussion of how this model was developed and what it offered that was considered new is needed. The probation license details are not discussed until p13 (lines 418-419), which is an omission. The initiative needs to be grounded on the broader policy landscape.

Therefore, the focus on housing needs to be clarified early on as p15 line 482 suggests that the model encompassed "housing, healthcare or employment services" suggesting a more holistic service than has been presented. The model needs to clarify what the practitioners could reliably do better that other through-the-gate initiatives could not. The focus included for instance harm reduction (for substance use). Did the caseworkers have training in substance misuse support, and if so, what level? Likewise, what training, expertise or support did the practitioners bring (were they housing specialists, for instance?). Had the caseworkers been offered support by housing services, if not, why not?

In that vein, the work of the practitioners needs further description. How do they choose, refer to, and access "community resources"? (e.g. p4 line 132) Was there an audit of available services, and were any bespoke referral mechanisms brokered by this model?Furthermore, the importance of engaging individuals was rightly emphasized. How did the team do this (motivational interviewing techniques, etc)? What was the skill base and experience of the team? How were they recruited for the role etc?

As suggested, the model needs to be clearly defined earlier on. We find on p8 a good description in the quote pp265-266, but the work should be clarified before that. Initially, I assumed the CTI worker would have privileged or some enhanced access to housing stock, but this doesn't seem to be the case. I was also unclear why affordability and landlord requirements were not considered beforehand. Registered Social Landlord restrictions would be a significant barrier and are mentioned first on p13 (line 371). I would assume that these barriers would be identified and potential solutions put forward. If not, then the model broadly has a role similar to other prison or CJS approaches that rely on referral, advocacy, and support. I can’t tease out the distinctive characteristics underpinning CTI in the paper's narrative.

I'd also suggest more information on the eligibility criteria of prisoners at release and the selection and referral processes to access the CTI.

How was at-risk of homelessness determined?

I feel the sole focus on caseworkers is a limitation. There is a need to provide alternative voices on the approaches deployed (for example, prison referral mechanisms, views of probation caseworkers, and notably, the perceptions of stakeholders in housing are crucial).

I'd suggest making it explicit throughout the document the actual numbers interviewed, which are 7 (p9, line 236).

I welcomed the discussion on supervision (p15), an area often neglected in the literature. Similarly, the point made on p16 about caseworker cohesion was well made. Given the complexity of the caseload, was sustainability considered in terms of reducing the likelihood of burnout, etc.?

The harm reduction section pp16-17 needs to be more explicit – what services are people referred to (treatment services, peer or mutual aid support), and how were these chosen? Was there an attempt to link with the recovery-orientated backing or just a focus on harm reduction? As mentioned, identifying problems with housing for people in areas with social problems is predictable – did the model consider mitigation strategies?

For the acceptability of the trials section, I would consider a separate paper or integrating some key points into a single paper. The section 'Wider Context' p23 is helpful, and some of the points should have been introduced earlier, as I've already stated (e.g., probation supervision arrangements). The points about the challenges of trials in a criminal legal setting will be well known to the audience (ethics, follow-up challenges) and the specifics of working in a criminal legal setting (e.g. Meade et al., 2018)

I struggled with the conclusion, which felt too vague, mainly due to the inclusion of the two components (process and RCT acceptability), which made it lose focus. The model has challenges that were not fully explored, and CTI in this paper, felt reactive, which may not be entirely an adequate representation of the model (e.g., affordability, landlord requirements, and moving to areas with high social problems that may worsen the likelihood of reconviction). Consideration of developing this model needs to be included, including how to leverage resources to address the problems identified. I think there are some valuable points about developing this model in a UK context that could be further teased out.

**Reviewer**	**2**
**Name**	**Lennox, Charlotte**
**Affiliation**	**University of Manchester, Manchester Academic Health Science Centre**
**Date**	**21-Feb-2025**
**COI**	**None**

Many thanks for the opportunity to review this article. It is well written and easy to read. I have recommended major revisions and highlighted a number of areas in the review checklist that are currently missing, but this all relates to the same issues. At the moment the process evaluation research questions and how data collected maps onto those research

questions to answer them (or not) is currently insufficiently described. The results are then not set out to answer these questions and there seems to be a lack of distinction between wider trial issues (perhaps best for the main trial paper) and here specifically on the process evaluation.

Below I set out areas that the authors may wish to consider.

Introduction

The statement that CTIs effectiveness for people leaving prison in the UK is unclear – does not perhaps summarise the evidence completely for example Continuity of care for recently released prisoners with mental illness: a pilot randomised controlled trial testing the feasibility of a Critical Time Intervention - PubMed and Critical time Intervention for Severely mentally ill Prisoners (CrISP): a randomised controlled trial - PubMed showed benefit for health related outcomes for people leaving prison in the UK.

The paper states that the primary aim was to conduct a pilot RCT, but this article focuses on the findings of the process evaluation. Would it be better for the aim of this paper to be the process evaluation? – perhaps some of the introduction needs to be given to providing a rationale for the benefits of process evaluations running alongside trials.

Also, what were the research questions, aims, hypotheses for the process evaluation – these need to be included to understand the methodological and analytical approaches for the process evaluation.

Methods

There seems to be quite a bit of unnecessary detail about the PHaCT trial in the summary section that is not really needed to understand the process evaluation, for example what the trial data collected, how it was collected and by whom etc. This could all be removed to allow more detail on the process evaluation.

The section called 'Process evaluation' could be largely redundant if the authors ended the introduction with a clear aim and research question for the process evaluation – this section feels overly wordy and duplicates content. Please amend.

Interview Participants

What was the total number of intervention delivery staff available to interview? What was the sampling strategy for the process evaluation interviews? Did you interview at one time point or multiple? Was it only CTI caseworks or did you interview other staff linked to the delivery of CTI?

It mentions that the intention was to interview trial participants – assume that this was to test the acceptability of trial methods? But it is not clear due to there being no clear aims or research questions for the process evaluation – how many trial participants did you aim to get, what was your sampling strategy for this? Even though this was not achieved (this point

can be talked about in the results and discussion and added as a significant limitation of the process evaluation) it still need to be set out in the method of what your intention was.

Observations

It would seem that only some of the fidelity checklist data is then presented in the results – this makes it quite difficult to follow and a suggestion would be in the method to only describe the data collection that is then used in the analysis for the process evaluation.

How many observations were planned? How did you decide which sessions to observe, how did you sample? Did this include all four sites, a range of practitioners etc?

It states that fieldnotes were collected, what were the focus of these notes, were they directed at particular aspects of delivery or a particular research question?

For clarity it would help to have a distinct section for the analysis setting out how each data pot (interviews, fidelity observations, fieldnotes observations) were individually analysed and then how the data were brought together and synthesised.

What was the studies analytical approach – it talks about themes and sub-themes, but also framework? The analytical approach is unclear.

The method section would benefit from a restructure, adding in clear research questions and then details methods of how each research question was to be tested.

Results

Out of 34 recruited – who? Is this staff or trial participants? It is not clear. Perhaps Out of the 34 trial participants recruited, 28 consented to be interviewed. However, only one… why was only one available?

7 interviews with CTI caseworkers – out of how many possible? Did all say yes? Did any interviews not take place?

135 hours for the observations – is that for the two researchers combined? Out of how many hours planned? The number of hours is not that informative, what would be more informative to show is how the observations were spread over CTI caseworks and CTI phases and sites. Without this level of detail it is very hard to assess the robustness of the evaluation data collection.

It would really help to have the results structured in relation to the aims or research questions e.g.

• Acceptability of the intervention

• Acceptability of trial methods

• Fidelity to the intervention

Without this structure it is really hard to understand and follow the decision making in terms of the themes that are presented.

The ‘theme names’ how do they relate to the research questions e.g. housing-led approach – is these theme providing evidence of the acceptability of the intervention or fidelity of the intervention?

If the authors want to present the results comparing and contrasting the 4 sites, then there needs to be more detail in the method to describe each site (the context of each site) and then within the data collection how much interviews and observations were spread over the 4 sites.

The sections in the results are very long and so I would suggest the authors select one quote per point. Also ‘CTI caseworker, site 1’ – is this always the same person quoted or could there be different CTI caseworkers at site 1? If so, it would be useful to give them all ID numbers.

The results seem to be dominated by site 1 and site 2 – why is that? Where there 2 sites but in each site 2 prisons – so 4 prisons in total?

I understand that there will be data presented in the main trial write-up that will focus on aspects of intervention delivery, but to understand the process evaluation some of that detail is also needed in this paper e.g. how much of the intervention was delivered to all participants, when participants ended the intervention, how many disengaged etc – this helps to understand the acceptability and fidelity to the intervention.

Acceptability of trials

This section would be useful to frame within the fact that the staff were already delivering CTI before the trial and therefore this is different to trials where CTI is a new offer – in this case equipoise is often not an issue and randomisation is acceptable, both to staff and participants. I wonder what the research team did in advance of the trial to support the sites to understand the value of randomisation and the need for a trial design – it would be helpful to include this information. Being involved in a current trial of an intervention that was already being delivered in some sites, we have had to undertake a significant amount of training and support for the intervention deliverers from them to understand this and to work with them to try and not undermine the randomisation. Did the research team consider cluster trial design, waiting list control etc – it would also seem that the control group offer was different – why was this, why was not the control offer standardised? If the aim of the process evaluation is to understand the acceptability of the trial methods, then the trial methods under examination need to be presented in the methods. The unhappiness of the staff to randomise seems completely understandable, if no work was done to support them changing their practice to deliver CTI in a trial design – therefore the unacceptability is not of trials per se but of the team not to anticipate this in advance. Likewise, the issues of acceptability resulting in differences between the control groups, why was this not addressed by the research team in study set up – again this could have been reasonably hypothesised as a risk to the trial.

The nature of informed consent

Was this an aim of the process evaluation to look at consent? If so this needs setting out in the method with an explanation of what data would answer this question.

What processes did the research team put in place to ensure a robust consent process? Who explained the study to participants, how well was the information adapted for the literacy and comprehension levels for people in prison, did the PPI members help with developing explanations of randomisation? Where these tested before the start of the trial? Did the research team work with the sites to support the staff to help them explain research concepts?

In the discussion it states participants fully comprehended the study's aims and procedures how do you know this? How did you test for comprehension. We worked to tailor communication strategies and included additional support to facilitate understanding such as having a randomisation script – could this be added into the method?

In this study as the same team are delivering both trial arms, was that explained to participants? It would be easy to anticipate confusion in this case – different in trials where the intervention is delivered by a different team to the control group – did the research team anticipate this? This is not necessarily as issue with consent in all prison trials but unique to those where there is no team separation in delivery.

The ethical concern around the control group is unclear – I am not sure that this is an issue about consent. This is for the research team to embed a robust process to ensure follow-up – so again, why was this not anticipated before?

Trial -specific challenges and reflections

How do these fit into the aims of the process evaluation? Who's reflections? Is this from the interviews? Or the research teams reflections? These seem to be core trial issues and are perhaps better covered in the main trial paper and in the discussion.

As outlined in the associated article (Line 687) there is no reference?

Wider context

As above this feels like it is a wider project issue and needs to be covered in the main trial and not focused on the process evaluation specifically.

Overall, the results section is rather confused. It is likely that this stems from the process evaluation aims, research questions and methods not being clearly set out. The authors perhaps need to rework the results, once these are set out so that the results presented are focused on the process evaluation (only).

Discussion

The discussion section will need revisions based on the comments suggested to the method and results.

It requires a strengths and limitations section (specifically around the process evaluation, based on what was planned vs what was actually done). In addition, I think an open and transparent discussion that some of the decisions made by the research team impacted the acceptability of the trial and issues around consent. From personal experience of prison trials, the use of CRN staff to deliver vital trial aspects such as recruitment and participant data collection, was a significant flaw. This trial and the decisions made by the team need to be contrasted with other prison trials, that have had dedicated data collection by researchers experienced in prison research, who worked hard to build trust and rapport to reduce attrition etc. e.g. Byng et al 2023. The authors state that 'as standard recruitment and follow -up methods often fall short with this population' – this needs some clarity as what the team attempted here might be standard in general population research, but it not standard for prison research, and some may argue were always going to cause issues. Therefore, some reflection of the experience of the research team in delivering prison trials previously would help.

VERSION 1 - AUTHOR RESPONSE

Reviewer comments	Authors response
**Reviewer 1**	
Introduction –<br>1.1 Expand the explanation of the CTI model. Clarify what practitioners do better than other through the gate initiatives.<br>Detail the training, expertise, and support provided to practitioners, especially in substance misuse support.<br>Describe how practitioners choose, refer to, and access community resources.<br>Differentiate the CTI model from other prison or CJS approaches.<br><br>1.2 Be clear that the location is England and Wales for an international audience.	1.1 Further detail on the CTI model was added to the section, see Housing-led Critical Time Intervention. We have briefly addressed each point, but we are unable to provide some of the detail required as it would not fit within this paper, and some of the requests are unable to be addressed as the Charity that delivers the intervention has been anonymised. Further explanation and detail of the model development would identify them.<br><br>1.2 Location made more explicit in the introduction (line 116-122).
Methods –<br>1.3 Provide more information on the eligibility criteria of prisoners at release and the selection and referral processes.<br><br>1.4 Explain how at-risk of homelessness was determined.<br><br>1.5 Include alternative voices, such as prison referral mechanisms, probation caseworkers, and housing stakeholders.	1.3 Trial eligibility criteria mentioned at the end of the introduction (138-144).<br><br>1.4 Added to section above.<br><br>1.5 We are unable to provide alternative voices as the groups mentioned were not interviewed.

1.6 Make the number of interviews explicit.	1.6 Added, see interview section (154-163).
Results –<br>1.7 Discuss supervision and caseworker cohesion and consider sustainability to reduce burnout.<br><br>1.8 Be explicit about harm reduction services and their selection.	 1.7 We have added a nod to burnout, but this was not prominent in our findings.<br><br>1.8 Examples were added to the harm reduction section (537 onwards).
Discussion –<br>1.9 Discuss engagement techniques like motivational interviewing and the skill base and experience of the team.<br><br>1.10 Consider a separate paper for the acceptability of trials or integrate key points into a single paper.<br><br>1.11 Introduce points about probation supervision arrangements earlier.<br><br>1.12 Explore challenges and development of the CTI model, including leveraging resources to address identified problems.<br><br>1.13 Refine the conclusion to avoid vagueness and maintain focus.	 1.9 This was not prominent in our findings and thus not a focus of discussion.<br><br>1.10 Combined with comments from reviewer 2, we have restructured/re-written the results and discussion to better integrate all findings.<br><br>1.11 Added to end of methods for context (215-227)<br><br>1.12 This paper focuses on the evaluation of delivery of the trial and intervention, but we did not design the intervention to be able to explore its development.<br>1.13 Conclusion re-written.
**Reviewer 2**	
Introduction:<br>2.1 The statement that CTIs effectiveness for people leaving prison in the UK is unclear – does not perhaps summarise the evidence completely for example Continuity of care for recently released prisoners with mental illness.<br><br>2.2 Better explain the aim of the process evaluation, not RCT as a whole	 2.1 The introduction section has been made clearer that evidence for specifically housing-led CTIs is unclear.<br><br>2.2 The aims section has been re-written to focus on the process evaluation with references to the RCT reduced.
Methods:<br>2.3 Remove the trial summary.<br><br>2.4 Amend the process evaluation section to better explain the evaluation.<br><br>2.5 Amend interviews and observations section	 2.3 The trial summary section has been removed. Shortened to a brief section at the end of the introduction for context.<br><br>2.4 The process evaluation section is amended as suggested.<br><br>2.5 interviews and observations amended as suggested

2.6 Restructure the methods section to focus on the evaluation.	2.6 The methods section was restructured as suggested.
Results:<br>2.7 Restructure the findings to match the evaluation aims, consider if the trial-specific challenges and wider context belong in this paper.<br><br>2.8 Reduce the length of the results section by having only 1 quote per point.<br><br>2.9 Update quotes reference to be a PID<br><br>2.10 Add context to the acceptability of the trial section	2.7 Large sections of the results rewritten and structured to follow the evaluation aims.<br><br>2.8 Multiple quotes removed to reduce length.<br><br>2.9 Quotes updated with PIDs.<br><br>2.10 Context added to the acceptability of the trial; see the acceptability of the trial design section
Discussion:<br>2.11 Revise the discussion based on changes from methods and results<br><br>2.12 Discuss some of the decisions made by the research team and the impacts of these.<br><br>2.13Add a strengths and limitations section	2.11 Discussion re-written based on changes to the results<br><br>2.12 Added to discussion.<br><br>2.13 Added strengths and limitations section

VERSION 2 - REVIEW

Reviewer 1
Name Sondhi, Arun
Affiliation Therapeutic Solutions
Date 10-Jul-2025
COI

Thank you for addressing the comments, and I'm comfortable with the responses. I would add my comment 1.5 as a potential limitation of the study.

Reviewer 2
Name Lennox, Charlotte
Affiliation University of Manchester, Manchester Academic Health Science Centre
Date 27-Jun-2025

COI

I would like to thank the authors for a very detailed response to the previous comments and for dealing with them in such a thorough way. I have no further comments to make and recommend this paper is accepted.

VERSION 2 - AUTHOR RESPONSE

Reviewer 1 - I would add my comment 1.5 as a potential limitation of the study.
Response: Added to limitations: "Additionally, the absence of perspectives from prison referrers, probation caseworkers, and housing stakeholders limits our understanding of how acceptable the intervention is to those responsible for making referrals." Page 21.
